# Mapping the epitopes of *Schistosoma japonicum* esophageal gland proteins for incorporation into vaccine constructs

**DOI:** 10.1371/journal.pone.0229542

**Published:** 2020-02-27

**Authors:** Xiao-Hong Li, Gillian M. Vance, Jared Cartwright, Jian-Ping Cao, R Alan Wilson, William Castro-Borges

**Affiliations:** 1 National Institute of Parasitic Diseases, Chinese Center for Disease Control and Prevention, Shanghai, People’s Republic of China; 2 Centre for Immunology and Infection, Department of Biology, University of York, York, England, United Kingdom; 3 Protein Production Laboratory, Department of Biology, University of York, York, England, United Kingdom; 4 Departamento de Ciências Biológicas, Universidade Federal de Ouro Preto, Campus Morro do Cruzeiro, Ouro Preto, Minas Gerais, Brasil; University of Texas at El Paso, UNITED STATES

## Abstract

**Background:**

The development of a schistosome vaccine has proved challenging but we have suggested that characterisation of the self-cure mechanism in rhesus macaques might provide a route to an effective product. The schistosome esophagus is a complex structure where blood processing is initiated by secretions from anterior and posterior glands, achieved by a mixture of ~40 unique proteins. The mechanism of self-cure in macaques involves cessation of feeding, after which worms slowly starve to death. Antibody coats the esophagus lumen and disrupts the secretory processes from the glands, potentially making their secretions ideal vaccine targets.

**Methodology/Principal findings:**

We have designed three peptide arrays comprising overlapping 15-mer peptides encompassing 32 esophageal gland proteins, and screened them for reactivity against 22-week infection serum from macaques versus permissive rabbit and mouse hosts. There was considerable intra- and inter-species variation in response and no obvious unique target was associated with self-cure status, which suggests that self-cure is achieved by antibodies reacting with multiple targets. Some immuno-dominant sequences/regions were evident across species, notably including: MEGs 4.1C, 4.2, and 11 (Array 1); MEG-12 and Aspartyl protease (Array 2); a Tetraspanin 1 loop and MEG-n2 (Array 3). Responses to MEGs 8.1C and 8.2C were largely confined to macaques. As proof of principle, three synthetic genes were designed, comprising several key targets from each array. One of these was expressed as a recombinant protein and used to vaccinate rabbits. Higher antibody titres were obtained to the majority of reactive regions than those elicited after prolonged infection.

**Conclusions/Significance:**

It is feasible to test simultaneously the additive potential of multiple esophageal proteins to induce protection by combining their most reactive regions in artificial constructs that can be used to vaccinate suitable hosts. The efficacy of the approach to disrupt esophageal function now needs to be tested by a parasite challenge.

## Introduction

Schistosomiasis remains a widespread and important public health problem in endemic regions, not least because widely available diagnostic methods are relatively insensitive [1]. While the intensity of infection has undoubtedly declined in many endemic areas due to mass treatment programmes using the drug Praziquantel [2], recent studies indicate that the number of low-intensity cases has been seriously and consistently underestimated [3, 4]. This diagnostic deficit is being addressed [5] but remains a major obstacle to the control and elimination of the disease. An effective schistosome vaccine would be an invaluable tool in this context [6, 7], especially against zoonotic schistosomiasis japonica. The power of vaccines to control and even eradicate viral and bacterial infections is evident, but development of vaccines against parasites has proved more challenging. The one schistosome vaccine that has undergone Phase III trials in school children, namely Bilhvax (rSh28GST), was immunogenic and well tolerated but did not cause a significant delay in the recurrence of urinary schistosomiasis [8]. Three other vaccines (SmTSP-2, Smp80, Sm14; [9]) are progressing through clinical trials and reports on their efficacy are eagerly awaited. However, they represent just a tiny fraction of the proteins exposed at, or released from, the parasite-host interface [10] so many other possibilities remain for potential exploitation in a vaccine.

The schistosome esophagus is a short tube, invested with longitudinal and circular muscles, responsible for conveying blood from the mouth to the worm’s intestine. However, the term esophagus is misleading since the structure is much more than just a conduction tube. The lumen is divided into anterior and posterior compartments by three smooth muscle sphincters [11]. A small anterior and a large posterior gland surround the two compartments into which they pour their secretions [12, 13]. During blood ingestion erythrocytes and platelets are lysed as they pass through the anterior and posterior compartments and leucocytes are also damaged, with some becoming tethered as a plug in the posterior lumen. All these processes are achieved by secretions from the glands, and a combination of RNASeq, in situ hybridisation and immunocytochemistry has been applied to their characterisation [12, 14]. These studies revealed that a mixture of approximately 40 unique proteins is responsible for initiating blood processing in the adult worm esophagus. The largest group comprises proteins encoded by micro-exon genes (MEGs), none of which have homology to proteins outside the Schistosomatidae. In addition, genes encoding a number of hydrolases, protease inhibitors, venom allergen like (VAL) and membrane structural proteins are differentially expressed in worm heads [14]. This molecular arsenal points to a series of highly specific interactions taking place in the esophageal lumen between the ingested blood and gland secretions. These processes effectively disable both the humoral and cellular attack mechanisms of the host, while preparing the blood constituents for hydrolysis by enzymes from the gastrodermis that lines the gut. The esophageal proteins represent a cohort of potential and hitherto unexploited vaccine candidates [12].

The rhesus macaque is unusual among hosts in that it can self-cure from a patent schistosome infection [15, 16]. Furthermore, the animals which have self-cured are fully resistant to a challenge exposure to cercariae [17–19]. In our recent investigation of a *S*. *japonicum* infection in the rhesus macaque we showed that faecal egg output peaked at 8 weeks but then declined rapidly towards zero by 18 weeks; the level of circulating antigens also declined but with a lag [15]. Surviving worms, recovered by portal perfusion at 22 weeks, were pallid and in the case of females reduced in size. Indeed it appeared that blood feeding had ceased and the worms were slowly starving to death, with host antibody implicated as the key agent. The mechanism of vesicle release in the anterior esophagus was disrupted, with large deposits of antibody coating the surfaces. The morphology of the posterior esophageal lumen was also modified, the lamellae being closely adherent, with antibody binding strongly to the luminal edge [15]. Given our estimate of at least 40 proteins secreted by the esophageal glands it is not an easy task to determine which constituents might be crucial in mediating protection. Indeed, multiple targets may be involved and, since mice make antibodies to esophageal proteins but do not self-cure, the intensity of the host response to key secretions may be equally relevant. The importance of the intensity of host response is reinforced by studies on light infections of rhesus macaques by both *S*. *mansoni* and *S*. *japonicum* where self-cure was not observed over long time periods [20, 21].

It is axiomatic that antigenic targets mediating protection must be directly accessible to immune effectors on the live schistosome or released into its immediate environs. The advent of proteomics has permitted the definition of cercarial secretions [22], the tegument surface [23–26], and indirectly, the gastrodermis via analysis of worm vomitus [27], especially in *S*. *mansoni* but also in *S*. *japonicum* [28]. In turn this has allowed the construction of targeted protein arrays to screen for likely immune targets [29, 30]. The serum obtained at 12 and 22 weeks from our *S*. *japonicum* experiment in rhesus macaques was previously used against such an array containing 172 *S*. *japonicum* proteins predicted to be localised to the tegument [30]. Eight proteins were detected by the serum pool but the only notable finding was the reactivity with an extracellular superoxide dismutase of unknown localisation. Recently, peptide arrays have become commercially available (https://www.pepperprint.com/) allowing the mapping of individual epitopes within proteins, using overlapping 15mer peptides printed onto glass slides. In this study we report on the design of three such arrays encompassing 33 esophageal proteins from *S*. *japonicum* identified in our differential transcriptional profiling of males and females [14]. These arrays were screened with serum from self-curing macaques, chronically infected rabbits and mice to search for patterns associated with the self-cure response. We report on the heterogeneous reactivity of these sera with the arrays, and identify immunodominant regions. As proof of principle, the DNA sequences of several regions were combined in a synthetic gene, and its expressed protein was used to vaccinate rabbits, generating high antibody titres against the majority of regions. This approach may open a route to the simultaneous vaccination of hosts with a small number of multi-epitope constructs encompassing a large number of target proteins to test for additive effects in blocking esophageal functions.

## Materials and methods

### Ethics statement

Shanghai: Rabbit and mouse infection sera used in this study were generated at the Institute of Parasitic Diseases, Chinese Center for Disease Control and Prevention. Animal care and all animal procedures were carried out in compliance with the Guidelines for the Care and Use of Laboratory Animals produced by the Shanghai Veterinary Research Institute. The study was approved by the Ethics Committee of the Institute (ID SYXK 2016–00196). The rhesus macaque sera came from a previously reported study [15] at the Kunming Institute of Zoology, Chinese Academy of Sciences (CAS). The experimental protocol was approved by the Ethics Committee of Kunming Institute of Zoology, (ID SYDW-2011017).

York: Rat positive control sera were generated for previously reported studies [12, 15] authorised on personal (PIL 50/592) and project licences (PPL 60/4340) issued by the UK Home Office. The study protocol was approved by the Biology Department Ethical Review Committee.

### Source of self-curing, chronic infection and positive control sera

Cercariae of *S*. *japonicum* were shed from naturally infected *Oncomelania hupensis* snails collected from fields in Anhui Province, P.R. China. Four outbred New Zealand white rabbits and four groups of Swiss mice were infected percutaneously with 200 or 25 cercariae, respectively. At 22 to 24 weeks post-infection the animals were bled to provide individual (rabbit) or group (mouse) pools of sera for array screening. The rat positive control sera comprised antibodies against MEG-4.1 (three peptides), MEG-4.2, MEG-8.2, MEG-9, MEG-11, MEG-14, and recombinant VAL-7 [12, 15]. Mouse positive control sera against MEG-12 (one peptide) and Phospholipase A #1 (three peptides) were generated in Shanghai by coupling to carrier ovalbumin, formulation in Freund’s complete (prime) or incomplete (two boosts) adjuvant, and subcutaneous administration at three week intervals before a final bleed at 8 weeks. Mouse antiserum against rSjGST-26 was a gift from Mr Yu-Xin Xu, NIPD, Shanghai.

### Array design

Proteins for inclusion in the three arrays were selected on the basis of differential transcript expression in *S*. *japonicum* male and female heads versus tails [14], taking account of predicted signal peptides and regions of O-glycosylation. The signal peptide, where present, was excised as it would not appear in the mature proteins secreted from or expressed on the worm. Array 1 was initially printed with seven of the most abundant MEG proteins, based on transcript (4.1, 4.2, 8.1, 8.2, 9, 11 and 14), plus VAL-7 and an irrelevant non-esophageal protein (Array 1a). It was subsequently reprinted with the same MEGs plus a short fragment of VAL-7 and full length SjGST26 to serve as a mouse positive control (Array 1b). The central repeat region of MEG-4.1, predicted to be heavily O-glycosylated, was omitted as were the N-terminal regions of MEGs 8.1, 8.2 and 14. The logic was that if the native proteins were so decorated, then these peptide regions would not be directly accessible to immunoglobulins in an infected host. For the MEG proteins, the longest isoform was printed. While exon skipping generates amino acid sequences that might be neo-epitopes it is not feasible to print all possible combinations, so this seems the best compromise. Array 2 was printed with further MEG proteins of lower transcript abundance (8.3, 8.4, 12, 15, 19, 22, 26.1 and 29), and the enzymes palmitoyl thioesterase (PTE), aspartyl protease, phospholipase A (PLA) #1, and PLA #2. Array 3 was printed with four further members of the MEG-26 family, two putative MEG proteins, n1 and n2, the inhibitor cystatin, SjNatterin (a potential kininogen), and four probable membrane structural proteins, namely two annexins and two tetraspanins. Only the two external loops of each tetraspanin were printed. All proteins were printed as overlapping 15mers. For Array 1 the offset was one amino acid, except for SjGST26 with two to accommodate its larger size. Array 2 was printed with a three amino acid offset to accommodate the higher molecular weight of the four enzymes (32.1, 43.2, 44.6 and 44.5 kDa versus a mean of 8kDa for the 21 MEGs on the three arrays). Array 3 was printed with a two amino acid offset. Each array comprised a matrix of 20 x 68 unique features printed in duplicate, including space for the quality control peptides Flag (DYKDDDDKAS) and HA tag (YPYDVPDYAG). Each slide comprised five copies of the array for incubation in a 3x5 well incubation tray. The printed sequences are listed in S1 Fig

### Array screening and data analysis

Peptide Microarrays were screened according to the manufacturer PEPperPRINT’s protocol at www.pepperprint.com. Briefly, slides were blocked, pre-stained with relevant secondary antibody, washed, dried and scanned to identify pre-existing reactivities. They were then re-blocked, stained with primary antibody, washed, stained with secondary antibody, washed, dried and scanned. Lastly, the PEPperPRINT control antibodies were applied before a final scan. Variations from the currently recommended protocol were as follows: bovine serum albumin (Sigma, Poole, Dorset, UK; A7030) was used as the blocking agent in the buffer solutions at a dilution of 1% in the blocking buffer and 0.5% in the staining buffer; blocking solutions were filtered using Millex HA 33mm 0.45μm filters, and primary and secondary antibody solutions using Millex LG 0.2μm filters (Merck Millipore Ltd, Carrigtwohill, Co. Cork, Ireland). Macaque, rabbit, mouse and rat primary antibodies were applied at the dilutions indicated in the relevant Figure legends. Their binding was detected using the following Cy3 and Cy5 labelled secondary antibodies.

Macaque: Cy3 Goat anti-human IgG (Fc specific) C2571 at 1:200 dilution, from Sigma (Poole, UK).

Rabbit, mouse and rat: Goat anti-rabbit IgG (H+L), Cy3 A10520 at 1:400, Cy5 A10523 at 1:300; Goat anti-mouse IgG (H+L), Cy3 A10521 at 1:300, Cy5 A10524 at 1:500; Goat anti-rat IgG (H+L), Cy3 A10522, at 1:200, all from Thermo Fisher (Cramlington, Newcastle, UK).

The two control antibodies provided by PEPperPrint, pre-labelled with Cy3 (Flag; DYKDDDDKAS) and Cy5 (HA; YPYDVPDYAG), were used at 1:1000 dilution.

Five arrays per slide were each treated with 400μl (300μl for PEPperPrint controls) of the appropriate solutions per well, with slow orbital shaking. Blocking, secondary antibody and control antibody solutions were each incubated at room temperature for 30 minutes; primary antibody solutions were incubated overnight at 4C. If repeating a test on an array that had been previously blocked, the blocking time was reduced to 20 minutes. Arrays could be reacted with primary antibodies of two different host species, provided that the secondary antibodies used for detection of antibody binding were labelled with different fluorophores and did not cross-react with the primary host species. Arrays were scanned using an Agilent DNA Micro Array scanner with High-Resolution Surescan Technology (Agilent Technologies LDA UK Limited, Stockport, Cheshire; model G2565CA). The instrument has a dynamic range > four orders of magnitude. A screengrab of the Agilent image was taken for orientation purposes. The Agilent .tif file output for each array was then analysed using the PepSlide® Analyzer (PSA) software and results sent to a .csv file in Excel for subsequent processing. Any blemishes visible on the screengrab were manually edited in the spreadsheet. The mean background values were subtracted from the test values by the PSA software before an aggregate green or red foreground mean value was obtained from the duplicate cells. Heatmaps or linear plots were then made from the cell scores for each array to facilitate visual interpretations. The mean PSA scores for each position on the array allow comparisons of the intensity of reactive regions, within a host species.

### Synthetic gene construction and expression

Although the individual peptide scores for each cell on the arrays cannot be directly compared between host species, they do provide a qualitative impression of which peptides, and hence sections of the parent protein, are the most immunogenic in the schistosome-infected host. For zoonotic *S*. *japonicum* we chose to focus on universal epitopes across species, but the same exercise could be confined to a single host species such as the rhesus macaque if that host was to be the recipient of a synthetic vaccine. For each host species, to convert the visual impressions of reactivity provided by the heat maps (Figs 4, 5 & 6), the mean PSA scores across the arrays for a sequence of reactive cells representing a putative epitope or reactive region, were summed to give an overall score. The amino acids within this region, plus a margin either end then provided the sequence for incorporation into the synthetic gene.

In this way we tested the hypothesis that epitopes detected in esophageal proteins could be combined in artificial constructs to act as multi-epitope immunogens that would elicit antibodies when administered to known *S*. *japonicum* hosts. The coding DNA sequences for the selected epitopes were combined to create a synthetic gene (S2 Fig) which was codon-optimised for expression in *E*.*coli* using Optimizer (http://genomes.urv.es/OPTIMIZER/) with a Guided Random distribution. The gene synthesis was provided by Eurofins Genomics UK (Wolverhampton, UK).

### Preparation of the expression vector

The pETFPP_41 N-terminal expression vector was prepared using pETYSBLIC [31] as a template for an inverse polymerase chain reaction (PCR) with the oligonucleotide forward and reverse primers, d(CGC GCC TTC TCC TCA CAT ATG GCT AGC) and d(GCC GCT GCT GTG ATG ATG ATG ATG ATG G), respectively, and KOD Hot-Start DNA polymerase (Novagen, Merck, Feltham, UK). The linear PCR product was treated with *Dpn*I (Promega, Southampton, UK) and gel-purified (Qiagen, Manchester, UK). The synthetic gene was then subcloned into the pETFPP_41 vector by amplifying the template DNA by PCR with oligonucleotide forward and reverse primers, d(CAT CAC AGC AGC GGC GAA AAG CTG ATT CAA TTC TTC GCA TAC) and d(TGA GGA GAA GGC GCG TTA GGA CAC AAC TGC GAC GTT TTG), respectively, with KOD Hot-Start DNA polymerase. The DNA was incubated in an InFusion (Takara Bio, Saint-Germain-en-Laye, France) reaction with pETFPP_41, the product was transformed into *E*. *coli*, XL1-blue (Stratagene, Agilent Technologies), and the sequence confirmed. The resulting construct yielded an N-terminal fusion of 6xHIS and the peptide epitope sequences.

For protein expression the pETFPP_41-EP vector was transformed into *E*. *coli* BL21-DE3 (Novagen). Overnight cultures containing lysogeny broth (LB) and kanamycin (30μg/ml) were inoculated from glycerol stocks and incubated at 37°C and 200 rpm. The cultures were then used to inoculate 600 ml of LB containing kanamycin (30μg/ml) before further incubation at 37°C and 200 rpm. At a culture OD_600nm_ of 0.6Au, IPTG was added to a final concentration of 1mM and incubation was continued for a further 3 hours. The induced cultures were divided into 4x150ml aliquots and centrifuged at 7,000*g* for 5 min at 4°C. The supernatant was discarded and the pellets were stored at -80°C until required.

Purification of the His-tagged protein product was performed by Ni^2+^-affinity chromatography using the following procedure. Frozen cell pellets were thawed and resuspended in lysis buffer (20mM sodium phosphate, pH 7.4, 300 mM NaCl, 1mM DTT) prior to disruption by sonication. A pellet was then recovered by centrifugation at 12,000*g* for 10 min at 4°C and washed twice by resuspending in lysis buffer and centrifuging as above. The washed pellet was then incubated in solubilisation buffer (50mM sodium phosphate, pH 7.4, 8M guanidine-HCl and 1mM DTT) by gently mixing at room temperature for 16h before diluting the solution with lysis buffer to give a final concentration of 6M guanidine-HCl. The solubilised protein was then centrifuged at 45,000*g* for 30 min at 10°C and the supernatant recovered.

For denaturing purification, the solubilised inclusion bodies were then further purified by loading the extract at a flow rate of 1ml/min by Ni^2+^-affinity chromatography using a HiTrap column (GE Healthcare, Little Chalfont, UK) previously equilibrated in Buffer A (50mM sodium phosphate, pH 8.0, 300mM NaCl, 6 M urea and 20mM imidazole). On completion of sample loading, the column was washed with 10 column volumes (CV) of Buffer A before a linear 20mM-500mM imidazole gradient elution of specific protein over 10 CV Buffer B (50mM sodium phosphate, pH 8.0, 300mM NaCl, 6 M urea and 500mM imidazole). Fractions (7ml) containing the His-tagged protein were confirmed by SDS-PAGE and the pooled protein concentration was estimated by A_280nm_.

### Immunogenicity testing

Two NZ outbred white rabbits were immunised with the recombinant multi-epitope protein by Envigo RMS UK Ltd (Belton, Leicestershire, UK). The protocol involved a pre-bleed and then subcutaneous administration of 100μg of the Ni column-purified protein in PBS, emulsified with Freund’s complete adjuvant for priming and Freund’s incomplete adjuvant for two boosters, all 14 days apart. The final bleed was 14 days after the last boost. The reactivity of the rabbit antibodies was then screened at 1:1000 dilution against Array 1a, which contained all the putative targets. Binding was detected using Cy5 labelled goat anti-rabbit antibody at 1:300 dilution.

## Results

### Positive control sera confirm specificity of array detection

**Fig 1:**
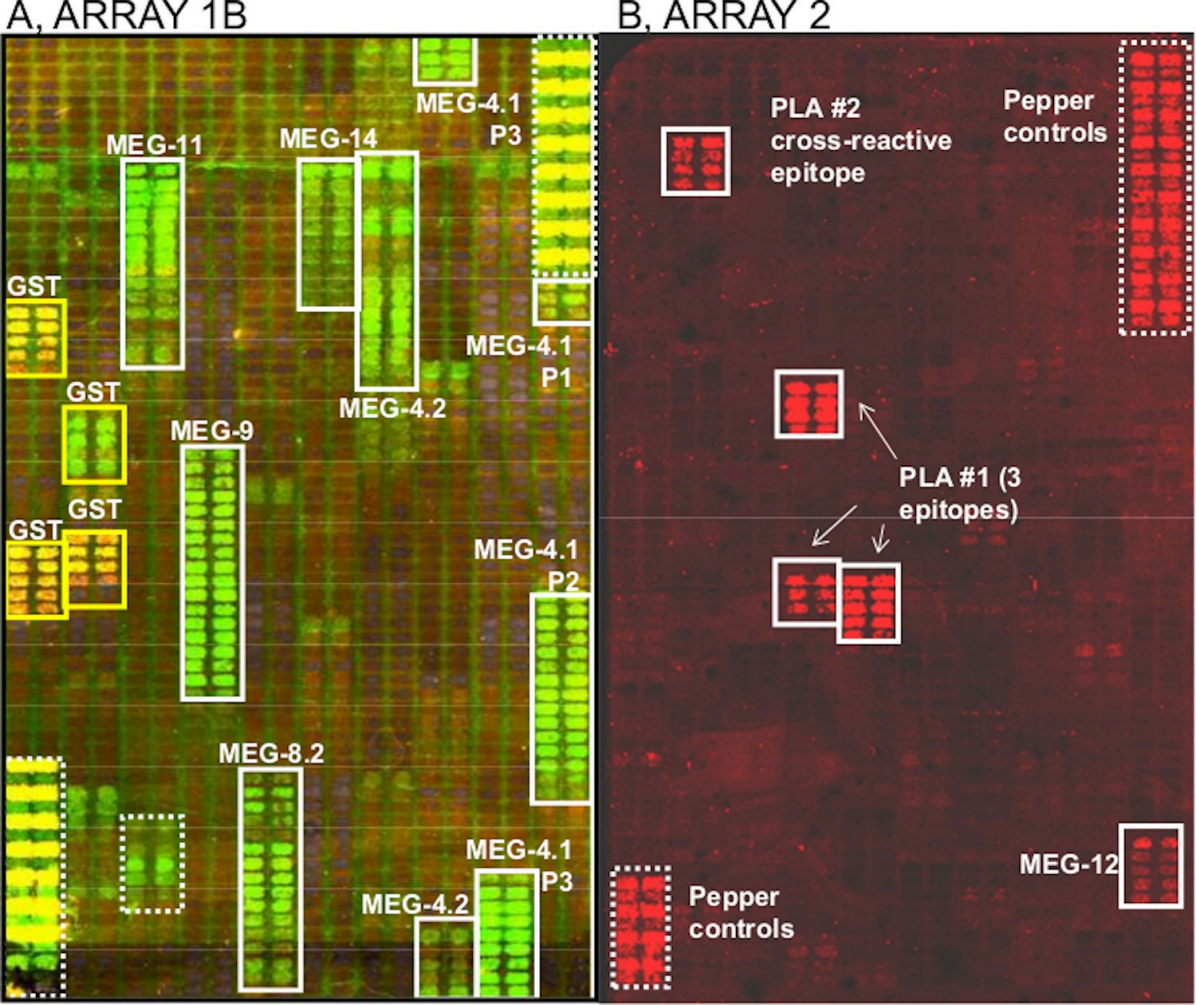
Screen grabs from the Agilent analyser showing the reactivity of positive control sera in annotated square boxes. The screen output is not a true representation of spectral emission lines but yellow/green in A corresponds to Cy3 at 570nm and red in B to Cy5 at 670nm. A, Reactivity of nine rat sera raised against defined peptide targets, applied at 1:1000 dilution, and mouse serum against recombinant Sj26GST, applied at 1:100 dilution, on Array 1b. The large boxes with dotted outline are the standard array controls supplied by PEPperPRINT. The small dotted box at bottom left delineates cells that reacted with the Cy3-goat anti-mouse prestain. All other cells were negative. B, Reactivity of four positive control mouse sera against peptides from MEG-12 and Phospholipase A #1 (PLA #1) applied at 1:2000 dilution, on Array 2, with Cy5 labelled secondary antibody. Antibody to MEG-12 had a relatively weak reactivity while the three anti-PLA1 sera detected epitopes of varying length and one (against PLA #1 peptide RGAPYDFRKSPDNK) also reacted with a related epitope in PLA #2.

**Fig 2:**
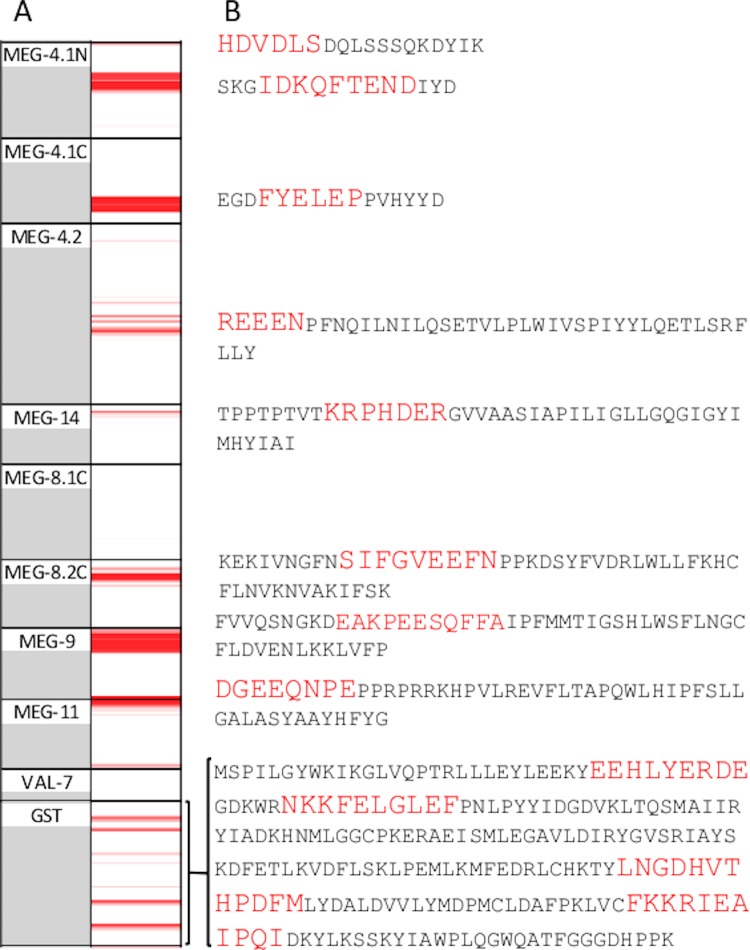
Heat map and peptides used for vaccination. Positive control A, Column 1 shows positions of 15mer overlapping peptides for the *S*. *japonicum* proteins printed on Array 1b. Column 2 shows the heat map of serum reactivity at 1:1000 dilution for rat and 1:100 for mouse, on a scale 1000–25000. B, Synthetic peptides used to immunise rats, and full length sequence of Sj26GST. Amino acids highlighted in red are the putative epitopes detected by the respective sera. No serum was available against MEG-8.1C.

The nine rat sera raised against the MEGs and VAL-7 in our previous studies on the worm esophagus confirmed the specificity of the epitope mapping approach by their reactivity with Array 1b (Fig 1A; Fig 2A). Each antiserum reacted with a sequence of array cells ranging from three (MEG-4.1N) to 18 (MEG-9) and the intensity of binding also differed markedly between the various MEG targets. The mouse antibody raised against recombinant Sj26GST revealed four major sites of reactivity each over five cells on the array, and some with lower intensity. Only anti-VAL-7 antibody failed to react with the fragment printed on Array 1b (or the full-length protein printed on Array 1a). One definite false positive was detected (dotted box, bottom left Fig 1A), as this reaction occurred after the pre-stain with goat anti-rat serum but before any primary antibody had been applied. A number of low-intensity reactions were also detected in single pairs of cells scattered throughout. The original peptides used to vaccinate the rats ranged in length from 15 up to 51 amino acids (Fig 2B), but it is clear from the number of cells detected by each antiserum that the reactive epitopes comprised discrete segments. These ranged in length from five to ten amino acids. MEG-4.2 appears to be an exception to this specificity in that bands on the heatmap outside the vaccinating sequence are also positive. Recombinant protein Sj26GST was printed with a two amino acid offset. This means that each of the four major epitopes detected by the vaccinated mouse serum spans approximately ten amino acids (Fig 2B).

**Fig 3:**
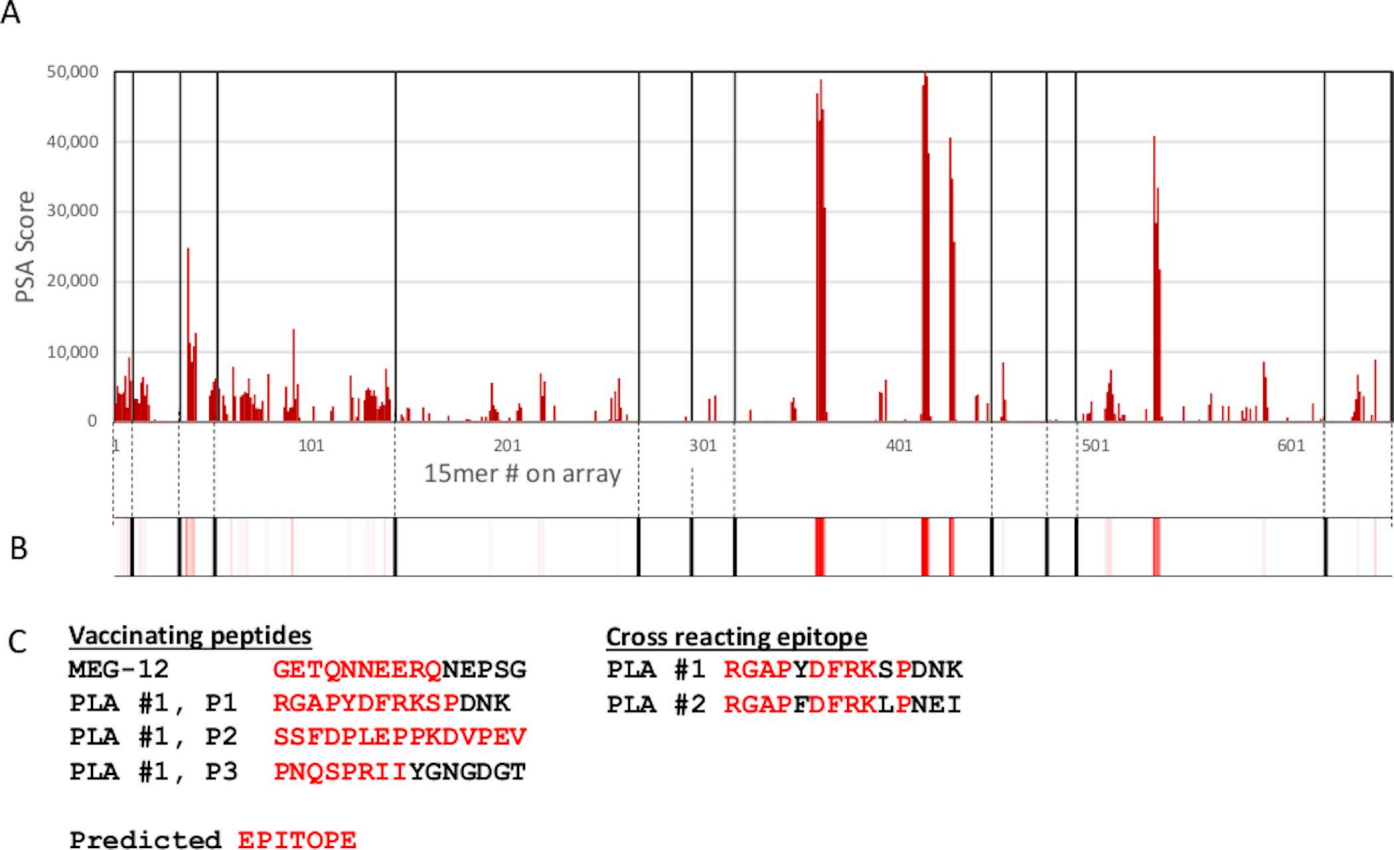
Positive control sera reacted with Array 2. A. Linear graphical plot showing reactivity of mouse antibodies at 1:2000 dilution against synthetic peptides from MEG-12 and Phospholipase A #1 (PLA #1). The x axis represents cell position on the array and the y axis the PSA score for each cell. B, corresponding heatmap on a scale 5000 to 50000. C. Peptide sequences used for vaccination of mice and a comparison of the cross-reacting epitope on PLA #1 and PLA #2. Note that a tyrosine and a serine in PLA #1 have been substituted by a phenylalanine and a leucine in PLA #2 with minimal effect on peptide reactivity.

Four sera raised in mice against synthetic peptides were also available as positive controls against targets on Array 2, one for MEG-12 and three for PLA #1. When reacted with the array, they all stained the expected cells (Fig 1B, screengrab; Fig 3A, linear plot; Fig 3B heatmap of Array 2), with moderate (MEG-12) to strong (PLA #1) binding. A single strong peak of binding was also observed in PLA #2. This cross-reactivity was explained when the sequence of the respective epitopes was compared (Fig 3C). There was a region of homology spanning 12 amino acids but with substitutions of a phenylalanine (F) for a tyrosine (Y) and a leucine (L) for a serine (S) in PLA #2. Clearly, there is some latitude in the binding specificity of an antibody to its epitope. The predicted size of the epitopes detected by the four antibodies was 10, 11, 15 and 8 amino acids respectively. Overall, the mean size of the 16 predicted epitopes mapped by the 12 positive control sera was 9.2 +/- SD 2.6 amino acids.

### Infection of macaques, rabbits and mice with *S*. *japonicum* elicits a heterogeneous pattern of antibody reactivities

**Fig 4:**
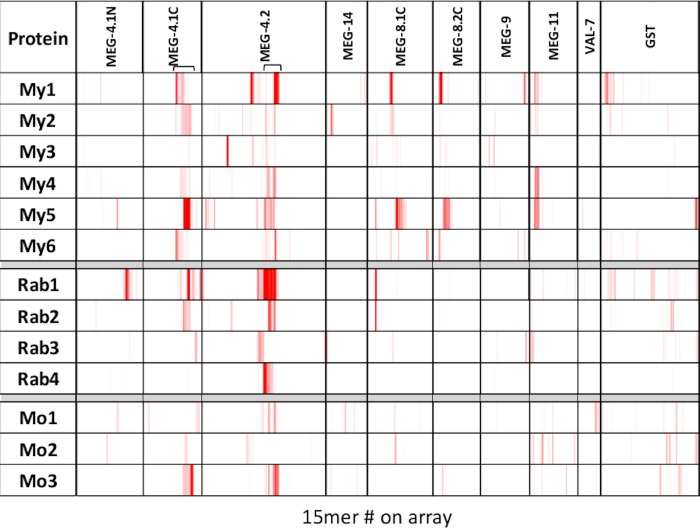
Heatmap of Array 1b. The array was reacted with six macaque (My) four rabbit (Rab) and three mouse (Mo) infection sera, all at 1:100 dilution. Location of the protein targets represented on the array by overlapping 15mer peptides is shown on the header. The heatmap is plotted on a scale of 1000 to 25000.

Inspection of the heatmap of the reactivities of macaques, rabbits and mice with Array 1b (Fig 4) shows a wide range within each host group. Clearly, a long-term infection of these hosts with adult *S*. *japonicum* produces a more complex pattern of reactivity than that observed with sera from rats or mice given single adjuvanted peptide vaccines (Fig 1). Focusing on the macaques, the N-terminus of MEG-4.1 shows only weak reactivity, whereas the C-terminus shows a broad band of common reactivity (bracketed), with only one weak reactor; MEG-4.2 has a similar broad band of reactivity (bracketed). The VAL-7 fragment is poorly reactive as is MEG-14, with only a single responder. MEG-9 is detected weakly by three macaques in two locations, whereas MEG-11 is detected by four and MEGs 8.1C and 8.2C by five responders, albeit with varying intensities. The self-curing macaques make a relatively poor response to Sj26GST, compared to those mice that had received a recombinant vaccine (Fig 1A).

With the four chronically infected rabbits, there are some similarities in the overall pattern, especially with MEG-4.2 and to a lesser extent MEG-4.1C terminus. One rabbit made a strong response to an epitope in the N-terminal region of MEG-4.1 while two rabbits made a response to the same region in MEG-8.1 C terminus (also seen weakly by three macaques). For the remainder of the array very little reactivity was observed, including Sj26GST, apart from rabbit 1. Two of three mice reacted with the same regions of MEG-4.1C and MEG-4.2 as the other hosts. There was only weak reactivity by the murine sera with the remaining proteins, apart from a moderate response by one animal to the VAL-7 fragment, weak reactivity to MEG-11 by a second, and with Sj26GST by all three.

**Fig 5:**
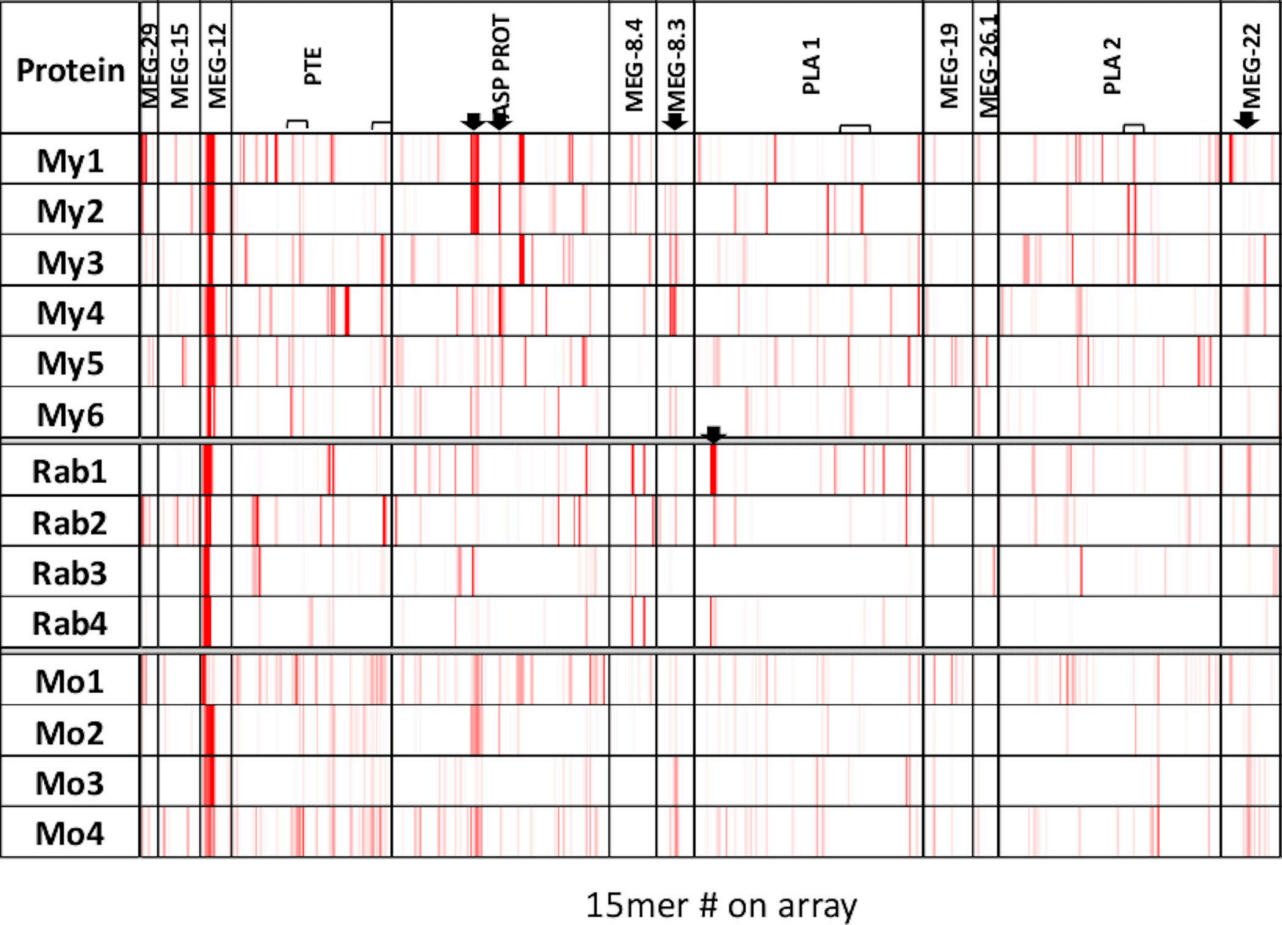
Heatmap of Array 2. The array was reacted with six macaque (My) four rabbit (Rab) and four mouse (Mo) infection sera, at 1:100, 1:200 and 1:1000 dilution, respectively. Location of the protein targets represented on the array by overlapping 15mer peptides is shown on the header. The heatmap is plotted on a scale of 1000 to 25000.

The proteins represented on Array 2 comprised eight MEGs and four enzymes, printed with a three amino acid offset. This is reflected in the apparent greater density of reactivities (Fig 5), compared to Array 1b (Fig 4), since there is three times more sequence coverage. There is again considerable variation in the patterns of reactivity between individuals in each of the three host groups. Considering the MEGs first, the most striking observation is the region of strong reactivity in MEG-12, apparent in all animals in the three groups. Indeed, MEG-12 appears to be the exception in that post-infection responses are stronger than those after vaccination with a peptide construct. Since the peptides recognised in the two situations are virtually identical, it must depend on the way they were presented to the immune system -possibly the duration of exposure. Other strong reactivities are confined to one or two individuals within a host group. Five of six macaques, two rabbits and four mice reacted with common regions of MEG-8.3 and MEG-22 (arrowed), at varying intensities. For the remaining MEGs, there were few commonalities within or between the host species. The patterns of reactivity with the four enzymes are equally complex; all are immunogenic but the epitopes detected vary between individuals. For PTE, two weak regions (bracketed) are detected by five of six macaques, but only one rabbit and two mice. There is a strong reaction by two macaques to a region in aspartyl protease (arrowed), to which the remaining four animals respond weakly; two rabbits and four mice respond to the same region. A second region in aspartyl protease is also detected weakly/moderately by all macaques and three mice, but no rabbits (arrowed). The within-species heterogeneity of response is most marked against the two phospholipase A enzymes. The strongest reaction is by a single rabbit to a region of PLA #1 (arrowed), weakly seen by two other rabbits and one macaque. There are two weak reactive regions (bracketed) common to most macaques in PLA #1 and PLA #2. The first is seen by no other animal and the second by one mouse.

**Fig 6:**
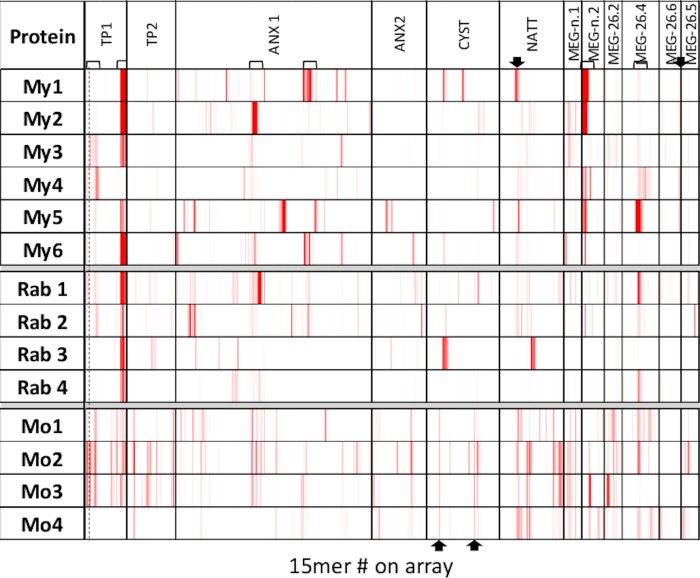
Heatmap of Array 3. The array was reacted with six macaque (My) four rabbit (Rab) and four mouse (Mo) infection sera at 1:100, 1:200 and 1:600 dilution, respectively. Location of the protein targets represented on the array by overlapping 15mer peptides is shown on the header. The heatmap is plotted on a scale of 1000 to 25000.

The proteins represented on Array 3 comprise six further MEGs, four membrane-associated proteins plus cystatin and SjNatterin. Among the MEGs, the strong reaction of four macaques to the unclassified MEG-n2 is evident (bracketed), plus weak reactions by a further macaque and two rabbits (Fig 6). One strong and three weak responses to MEG-26.4 (bracketed) are made by the macaques, plus three rabbits and three mice. Four macaques, but no other animals, made a weak response (arrowed) to the C terminus of MEG-26.6. Five macaques, four rabbits and three mice responded, mostly with a strong reaction, to the C terminus of the large Tetraspanin 1 loop (bracketed). The situation at the N-terminus of Tetraspanin 1 is complicated as it is a hybrid structure between the small and large extracellular loops. Two macaques, one rabbit and two mice made a weak response to the small loop while four macaques, two rabbits and three mice made a weak response to the N-terminus of the large loop (bracketed together). In contrast, only the mice reacted with some consistency to Tetraspanin 2. There were strong reactions by individual animals to different regions of Annexin 1 but no common reactivity. Two areas of generalised reactivity with varying intensities (bracketed) between most animals were therefore noted. No obvious common responses were observed between species for the partial Annexin 2. Although the mice made a weak response to two cystatin epitopes (arrowed), these were not detected by macaques or rabbits. Finally, a response was made to one region of Natterin by five macaques, one of them strongly (arrowed); two rabbits and all mice also reacted in this region.

We anticipated that epitope mapping of the most prominent esophageal proteins might pinpoint those epitopes associated with self-cure. On a qualitative basis, no obvious group of targets leap out when macaque responses are compared with those of rabbits and mice. The PSA scores within species provide a quantitative estimate of reactivity with the parent esophageal protein. We only have worm burdens for the 6 rhesus macaques [15], with worm recovery varying from 15 to 116, but the physiology of virtually all surviving worms was severely impaired. Whichever way the data were interrogated, using total PSA scores or those of the most reactive regions (S3A Fig), there was no positive correlation with worm burden (P>0.05). Indeed macaque #1 with the highest PSA score had 110 surviving worms at the 22 week perfusion, so confounding any statistical test. Previously, we tested the reactivity of these same macaque sera against esophageal gland targets by standard ELISA [15]. Not too much can be read into a comparison with the array PSA scores due to differences in targets. In five out of seven cases the ELISA plates were coated with a single 15mer peptide coupled to carrier BSA whereas on the array each protein was completely represented by overlapping 15-mers. Macaque #5 was the strongest overall reactor using both approaches while macaque #1 was weak by ELISA but the second strongest by array reactivity. Macaques # 4 and 5 were the strongest reactors with MEG-11 by both ELISA and array.

The proteins printed on the arrays were selected on the basis of differential RNA-Seq on male and female *S*. *japonicum* heads and tails [14], reinforced by almost identical RNA-Seq findings from *S*. *mansoni* [32]. On the premise that protein abundance might follow transcript abundance, we tested whether the reactivity of macaque serum with the printed peptides was related to transcript abundance in the esophageal glands. We used the mean PSA score for each protein as the measure of reactivity while transcript abundance was expressed as Fragments Per Kilobase of transcript per Million mapped reads (FPKM) [14]. The macaque sera were reactive with 22 out of the 32 gland products printed on the array. MEG-12 (FPKM, 9.7) and TP1 (FPKM, 6.5) were the most highly reactive proteins but not the most highly expressed transcripts. In contrast the highly reactive MEG-4.2 (FPKM, 12.6) and MEG-4.1 (FPKM, 12.4) were also highly expressed, but MEG-9 (FPKM, 12.1) showed poor reactivity. Some macaques responded to MEG 8.1 (FPKM, 9.6), MEG-8.2 (FPKM, 13.1) and MEG-11 (FPKM, 10.6) where there was little reactivity with rabbit or mouse sera. The enzymes had FPKMs between 4.9 and 7.2 and the MEG-26 family between 4.9 and 7.2. However, when the data were viewed as a whole, there was no correlation between PSA score and FPKM (scatter plot, S3B Fig). Clearly the immunogenicity of the esophageal gland products must be determined by other factors than predicted abundance in the secretions based on FPKM transcript score.

### Selecting epitopes for a synthetic gene

Seven reactive regions were selected from Array 1b for encoding in a synthetic gene (S3 Fig), in part because all seven were amongst the most abundant differentially expressed transcripts detected by RNASeq [14]. MEG-4.1C and MEG-4.2 had moderate to high scores in all host species (Table 1). MEGs 8.1C, 8.2C and 11 had moderate scores in the macaques, while MEG-9 had only a weak score in both macaques and mice. We also included a fragment for VAL-7, predicted by Bepipred, although negligible reactivity with VAL-7 peptides had been found on either Array 1a or 1b. Similar regions were selected for the most reactive proteins on Arrays 2 and 3 (S2 Table; S3 Table) although these were not taken further.

**Table 1:** Peptide selection for a synthetic gene.

Protein	Host	Score	Peptide for construct
MED-4.1C	My	90474	**EKLIQFFAYLVEEGDFYELEPPVHYYDYSV**
	Rab	56300	EKLIQFFAYLVEEGDFYELEPPVHYYDYSV
	Mo	46534	FFAYLVEEGDFYELEPPVHYYDYSVP
MEG-4.2	My	52774	**YEVLWTEELRPDIDKFHYDDTFRDFPRQKLREEENP**FNQ
	Rab	201309	YEVLWTEELRPDIDKFHYDDTFRDFPRQKLREEENPFN
	Mo	117705	WTEELRPDIDKFHYDDTFRDFPRQKLREEENPFN
MEG-8.1C	My	46229	**MQKINDGFFYLFSEQEFHPLHDKSYLFNIW**
	Rab	22123	**RKPKDVQQLGEKKTIM**
	Mo		
MEG-8.2C	My	51002	**EKIVNGFNSIFGVEENPPKDSYFVDRLW**
	Rab		
	Mo		
MEG-9	My	7222	DVENLKKLVFP
	Rab		
	Mo	2518	**DEAKPEESQFFAIPFMMTIGSHLW**
MEG-11	My	34292	**DGEEQNPEPPRPRRKHPVLREVFLT**
	Rab	9094	DGEEQNPEPPRPRR
	Mo	22985	DGEEQNPEPPRPRRKHPVLREVFLT

The construct is based on the reactivity of all sera with Array 1b. The score colour-coded in shades of red is the mean value for each reactive 15mer peptide across each host group, totalled for the region of reactivity, as shown in the heatmap in Fig 4. Note that some regions of reactivity may span two or more putative epitopes.

Purification of the recombinant protein expressed in *E*. *coli* was performed from the equivalent of 150ml of LB culture. Cell lysis was achieved by sonication and purification was performed from inclusion bodies using denaturing Ni2+-affinity chromatography before dialysis. Purity was determined in SDS-PAGE under reducing conditions revealing greater than 99% for the product at approximately 27 kDa, which corresponds well with the predicted mass of 27,066 Da (S4 Fig). The total yield was 7.65 mg at a concentration of 0.85mg/ml, as a precipitate in PBS.

**Fig 7:**
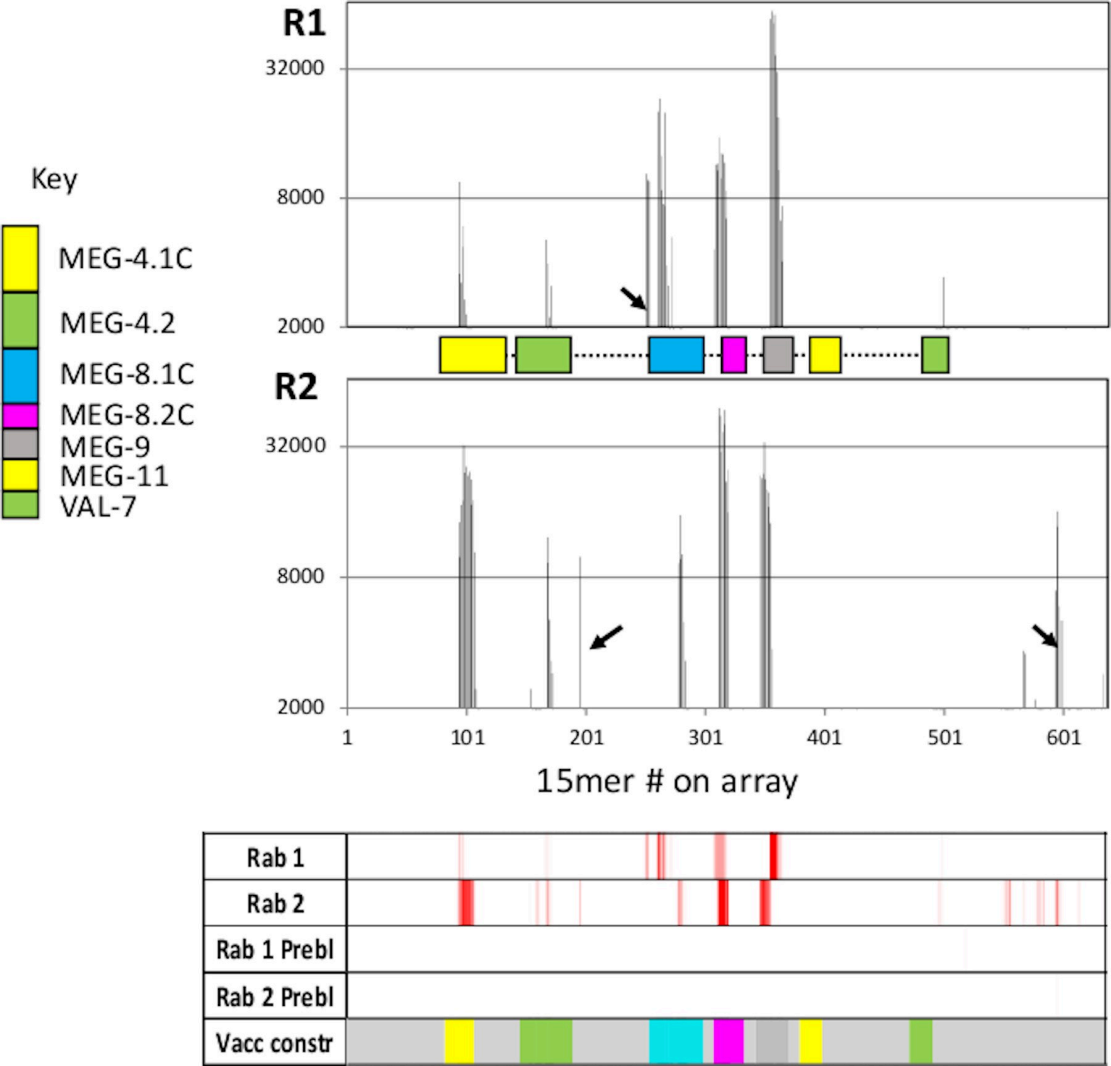
Reactivity of the recombinant protein comprising seven regions of the proteins represented on Array 1a. Linear plots and corresponding heatmap show that five of seven constituent peptides elicited a response in two outbred rabbits (MEG-11 was the notable exception). The primary antibody was reacted at a dilution of 1:1000; the intensity of response revealed by the PSA scores is much higher than after infection of rhesus macaques, rabbits or mice with *S*. *japonicum*.

As a test of immunogenicity, two rabbits received three subcutaneous administrations of 100μg purified recombinant protein emulsified in adjuvant. Sera from the pre-immunization bleed and the final 8-week bleed were then analysed for reactivity against Array 1a (Fig 7). There was negligible reactivity in the pre-bleed serum but both rabbits responded strongly (but not identically) to epitopes in the artificial recombinant protein. For rabbit 1 the intensities were MEG-9>MEG-8.1C>MEG-8.2C>MEG-4.1C>MEG-4.2. There was no response to MEG-11 or the VAL-7 fragment and one false positive was detected at the boundary with MEG-8.2C (arrowed). For rabbit 2 the intensities were MEG-8.2C>MEG-9 = MEG-4.1C>MEG-8.1C>MEG-4.2. Again there was no response to MEG-11 or VAL-7 and two false positives were observed (arrowed).The reactive regions of MEG-8.1C in the two rabbits were distinct, with no overlap, while the other four regions were broadly similar.

## Discussion

### The array approach is specific and reliable

We confirmed the specificity of the array approach using sera raised in our previous studies of protein localisation in esophageal gland tissues [12, 15]. These 12 sera reacted with the anticipated regions of arrays 1 and 2 at varying intensities, with very little spurious binding. Indeed, it is worth noting that the intensity of the response after vaccination of rats/mice, with either peptide conjugated to carrier or recombinant protein, was generally more intense than that elicited after a schistosome infection, especially so for Sj26GST but with MEG-12 as a clear exception. The binding to between 3 and 18 contiguous cells on the array (cf. MEG-4.1N and MEG-9 on Array 1) provided an indication of linear epitope size. Our rough estimate of ~ nine amino acids fits the spatial arrangement of the Fab CDRs that make up the antigen binding site. It is also in line with a precise estimate arrived at by single amino acid substitutions in 79 linear epitopes derived from 22 PrEST human protein fragments: commonest length 7 to 9, range 4 to 12 amino acids [33]. This information is important when selecting regions of reactivity for inclusion in a multi-epitope construct.

### Merits and limitations of peptide arrays

A singular advantage of peptide arrays is that they provide a way to screen simultaneously and comparatively a large number of proteins for immunogenicity, at the fine specificity of the epitope. They allow regions of reactivity to be defined as linear sequences of amino acids that interact with the antigen-binding site of an antibody molecule. Whether portions of conformational epitopes might also bind antibodies is unclear. Some conformational epitopes contain short linear sequences as part of their more complex structure [34]. Our observations on epitope length (4–12 amino acids) suggest that antibodies may bind to part of an epitope represented within a 15-mer peptide. However, the complex epitopes showing widely spaced amino acids on e.g. virus capsids, mapped by high-resolution cryoelectron microscopy [35], are unlikely to be detected. We simply do not know how crucial are linear versus conformational epitopes in protective immunity to schistosomes. Esophageal secretions lack the rigid structure of virus capsids. Indeed, of the 21 MEG-encoded proteins printed on the arrays, 16 lack disulphide bridges while the other five possess a single pair of cysteines, implying all have a loose secondary structure. It is thus plausible that antibodies elicited by multi-epitope constructs comprising linear epitopes, which target the MEG proteins, may be adequate to block the feeding process of adult worms.

### The host sera identify epitopes in the majority of target proteins

The target proteins selected for the arrays form a tight cohort confined in expression to the anterior and posterior esophageal glands, as defined in our recent publications [12, 14, 15, 32]. The arrays were screened for total IgG binding, with no attempt made to determine subclass responses. This assumes that we are looking at the products of B lymphocytes requiring T cell help, since class switching has occurred. One caveat about our data is that quantitative comparisons between sera from the three host species reacted with the peptide arrays are not valid since different polyclonal antibodies were required to detect primary antibody binding. Our results show that it is possible to perform an in-depth screen using sera from three outbred host species to detect many reactivities against 32 esophageal gland proteins. In this respect the peptide array technology provided superior results to that of a full length *S*. *japonicum* protein array, where our macaque sera detected only 8 out of 172 targets [30].

Perhaps the most striking observation from our data was the large degree of heterogeneity in response between members of one species. This might be anticipated due to the extent of MHC polymorphism, and hence the range of peptides presented to the immune system. Ideally we would like to select universal epitopes for further consideration, especially in the case of zoonotic *S*. *japonicum*, which has many natural host species [36]. The proteins that stood out as having targets shared across species were: on Array 1, MEGs 4.1C, 4.2, and 11, with MEGs 8.1C and 8.2C largely confined to macaques; on Array 2, the N terminus of MEG-12 and two regions of the Aspartyl protease; on Array 3, the C-terminus of Tetraspanin 1 and the N terminal region of MEG-n2. The heterogeneity of response flags up a warning about the use of single antigen vaccines where, even if the candidate protein is protective e.g. in inbred mouse strains, almost inevitably there will be some non-responders in outbred populations. This is best exemplified by the hepatitis B surface antigen vaccine where 5–10% of healthy human recipients do not seroconvert [37].

In the case of MEG-4 and MEG-8 families, there is further information relevant to their potential role as vaccine candidates. Phylogenetic and structural analysis of MEG and VAL secreted proteins has shown high nonsynonymous/synonymous substitution (d*N*/d*S*) rates, as a result of evolutionary pressure from the host immune system [38]. In this context, our data revealed that VAL-7 peptides were poorly reactive with sera from the three infected host species and from rats vaccinated with recombinant VAL-7. Based on the absence of linear epitopes we are forced to conclude that VAL-7 was not a suitable component for a multi-epitope construct. Conversely, the regions of reactivity that were detected for MEG-4.1C and MEG-4.2 by serum from all three host species lie within the conserved C-terminal sequences of the two proteins in *S*. *japonicum*, *S*. *mansoni* and *S*. *haematobium* [12]. This level of conservation suggests a shared motif executing a common function in an interaction with host protein(s), which may be vulnerable to blocking by antibodies. A similar observation was made for the reactivity of macaque sera with the C termini of MEG-8.1 and MEG-8.2, and of macaque and mouse sera with the C terminus of MEG-8.3. Again, the antibody targets lay within the conserved sequence regions of MEGs 8.1 to 8.4 from *S*. *japonicum* and *S*. *mansoni* [32]. This reinforces a case for inclusion of the MEG-4 and MEG-8 family shared epitopes in a construct.

In our recent study of self-cure from an *S*. *japonicum* infection by rhesus macaques, we concluded that elimination of the adult worm population was the result of slow starvation due to cessation of feeding, with esophageal gland proteins as the most likely targets for antibody responses [15]. We undertook the mapping study with peptide arrays, to compare the macaque responses with those of mice and rabbits that do not self-cure, as an efficient and rapid way to screen samples at the fundamental unit of the antigenic epitope. The approach is very effective at identifying multiple targets in the three species, but qualitatively, we found no striking difference between macaques, rabbits and mice that would implicate single antigens as mediating the macaque self-cure process. Indeed, it was notable that most of the proteins on the arrays elicited an antibody response over the infection time course, in one or more animals of each species. These data suggest that multiple targets are involved in the protective response and that for the macaque, the intensity of response is also a key factor. Unlike the current experiment with *S*. *japonicum*, in our previous study with *S*. *mansoni*, the macaques did stratify into three groups: low, medium and high antibody responders [16]. Furthermore, since the anti-schistosome responses inversely correlated with worm burden at the 18 week perfusion point, those data support our conclusions about the importance of antibody intensity.

### Why focus on esophageal gland secretions?

What advantages might this cohort of esophageal proteins have as vaccine candidates? The first consideration is that unlike products of the tegument surface in contact with fast flowing blood, esophageal proteins act within the very confined space of the anterior and posterior esophageal compartments, (especially narrow in females). There, it is plausible to envisage disruption of biological function by the formation of immune complexes and the neutralisation of vital functions without the rapid dispersal of interacting components. On the basis of electron microscope evidence this is precisely what we observed in worms from self-curing macaques [15]. There, deposits of material appear to block pores on the luminal membrane of the anterior esophageal chamber, causing a build-up of secretory vesicles in the esophageal lining. Blocking the actions of proteins that disable ingested leucocytes could also enhance their survival and effector functions, to the detriment of the worm. A second advantage is that the esophageal secretions are distal to the hydrolytic lysosomal secretions of the gastrodermis. This may give time for ingested antibodies to interact with target proteins before they intermingle with gastrodermal proteases such as aspartyl proteases that may rapidly destroy them [39]. It could partially explain why MEG-12 from the anterior gland is a strong immunogen. The protein, suffering less proteolysis, is more likely to elicit a strong host IgG response when voided in vomitus and in turn those antibodies may neutralise their target *in situ* before they can be denatured.

### Knowledge of the epitope structure of esophageal proteins can be used constructively

Many early anti-microbial vaccines were simply killed organisms or crude extracts therefrom. With eukaryote parasites this approach has not worked [40] and while radiation-attenuated (RA) schistosome vaccines induce protection, they are not practicable. Consequently, investigators have searched for single antigen formulations with protective efficacy. We have questioned whether the ceiling of 50% attained in mice in such schistosome vaccine experiments is an artefact of the mouse model [41], but vaccine experiments with Smp80 calpain in baboons [42, 43] are not subject to that critique. Mathematical models suggest that at least 60% and preferably 80% reduction in egg output, inflicted by a vaccine, is needed to impact on schistosome transmission dynamics [44]. Perhaps the quest for a single antigen “magic bullet” vaccine has significant limitations? In evaluating the obstacles to successful immunisation against helminths, other researchers have pointed out that there is no reason to expect a single protein to be a sufficient target to disable a sophisticated parasite with a large number of coding genes [45]. Indeed it would be a remarkable weak spot if one could be identified. The two animal models where acquired immunity is demonstrable, the RA cercarial vaccine and the self-cure process in rhesus macaques, point to protection being mediated by multiple targets. In the case of the RA vaccine, the effector mechanism involves blocked migration of schistosomula in the pulmonary capillaries by an inflammatory response to worm secretions [46]. For the rhesus macaque, as outlined above, the protective mechanism involves a protracted immune pressure that leads to a slow decline in worm physiology and death from organ failure. If multiple targets mediate protection in these models then how do we identify them, and equally important how do we test their additive protective potential?

We hypothesised that it should be possible to use the epitope information gleaned from the array to generate an artificial “string of beads” protein for use in vaccination studies where the effector response would be directed to multiple targets. If successful, this would greatly reduce the number of recombinant proteins that needed to be incorporated in a vaccine directed against many targets. Our artificial protein contained seven regions of reactivity (one of them predicted, not actual) based on Array 1 data. Five of the constituents elicited antibody responses in the two immunised rabbits. An important question is why the MEG-11 peptide failed. Was this because it was immunogenic in macaques but not rabbits after a natural infection, so could be immunologically silent? Another possibility is that the artificial protein folded in such a way that the MEG-11 epitope was not exposed on the surface so was unable to interact with the membrane IgM antigen receptor of naïve B cells. We also showed that it would be feasible to make similar constructs based on Arrays 2 and 3. In this way we could combine the most reactive regions of ~30 proteins observed in vivo, into three recombinant proteins. Whether an admixture of such proteins could generate a strong antibody response to the components and whether that response was protective, needs to be tested by a parasite challenge. Certainly it represents the antithesis of the single magic bullet antigen approach.

## Conclusions

- The peptide microarray is a valid approach to define the antigenicity of individual proteins.
- It lends itself to the rapid processing of large numbers of esophageal proteins using multiple serum samples.
- The results reveal a large degree of heterogeneity in the response to those proteins between individuals and between host species, indicating that self-cure in the rhesus macaque is unlikely to be mediated by a single antigen.
- Nevertheless, comprehensive universal epitopes can be selected and combined into synthetic gene constructs for protein expression.
- When used to vaccinate a potential host, such proteins elicit a strong response against the majority of constituents.
- Extended in scale, it would be possible to vaccinate an animal with reactive regions/epitopes from 30 to 40 different esophageal proteins.
- Such procedures need to be tested in a suitable schistosome host by cercarial challenge.

## Supporting information

S1 Fig Protein sequences printed on the three arrays. (DOCX)

S2 Fig A. Lack of correlation between PSA scores and rhesus worm burden. B. lack of correlation between PSA score and transcript abundance.
(XLSX)

S3 Fig The artificial protein construct. A. The amino acid sequences are concatenated from Table 1. VAL-7 is represented by an epitope predicted by BepiPred. B, The nucleotide sequence of the synthetic gene for splicing into the expression vector, and its optimised version for expression in *E*. *coli*. C, Heat map of the reactivity of sera from two rabbits immunised with the recombinant protein, mapped onto Array 1a. The regions on the array represented by the protein are colour-coded.
(PPTX)

S4 Fig 1D gel of purified recombinant protein. (PPTX)

S1 Table A. Epitopes selected from Array 2. B. Concatenated amino acids for a putative artificial protein sequence.
(PPTX)

S2 Table A. Epitopes selected from Array 3. B. Concatenated amino acids for a putative artificial protein sequence.
(PPTX)

S3 Table Array data—Raw signal intensity data (mean of spot duplicates), acquired from the array experiments. (XLSX)
