# Supplementary material for: Mapping the epitopes of Schistosoma japonicum esophageal gland proteins for incorporation into vaccine constructs
Source: PLoS One. 2020 Feb 27;15(2):e0229542. doi: 10.1371/journal.pone.0229542 (PMC7046203; doi:10.1371/journal.pone.0229542)
Supplement: S1 Fig — (DOCX) [file pone.0229542.s001.docx]

**S1 Fig**

**SEQUENCES PRINTED ON ARRAYS**

All sequences are minus their predicted N terminal signal peptide where appropriate

Primary annotation is from Li et al PLoS Negl Trop Dis. 2018;12(2):e0006235.

In August 2019 [Luo F](https://www.ncbi.nlm.nih.gov/pubmed/?term=Luo%20F%5BAuthor%5D&cauthor=true&cauthor_uid=31390359), et al., published a paper describing an improved genome assembly for *S. japonicum*. (PLOS NTD Aug 7;13(8):e0007612. doi: 10.1371/journal.pntd.0007612), with data deposited on GenBank which has greatly improved publicly available annotations.

Depositions from the above project can be identified from the accession format: TNNnnnnn.1. Eight MEG-encoded proteins are still not represented on GenBank.

**ARRAY 1A**

>SjMEG-4.1, N-terminus. GenBank: TNN13460.1, CAX72670.1 and others

HDVDLSDQLSSSQKDYIKNKIRLLNDYFKSKGIDKQFTENDIYDLLNTRMNKHIQDKNIDIHIIHKKNETQPVR

>SjMEG-4.1, C-terminus. GenBank: TNN13460.1, CAX72670.1 and others

NYEKIDSLSKVFQLKKAFIPVWILNPLYYIIEKLIQFFAYLVEEGDFYELEPPVHYYDYSV

>SjMEG-4.2 (complete). GenBank: [CAX69761.1](https://www.ncbi.nlm.nih.gov/protein/CAX69761.1?report=genbank&log$=prottop&blast_rank=1&RID=X56E7ATX016)PGPYLGNEQSINLGKNDNQEHRKHKVATTPVHTMLHENVVNVSKRPEHSMPKYEVLWTEELRPDIDKFHYDDTFRDFPRQKLREEENPFNQILNILQSETVLPLWIVSPIYYLQETLSRFLLYLISRMW

>SjMEG-14, C-terminus. GenBank: [AAW25780.1](https://www.ncbi.nlm.nih.gov/protein/AAW25780.1?report=genbank&log$=prottop&blast_rank=1&RID=X56E7ATX016)

GNTSTHPTVTKRPHDERGVIAASIAPIILGLLGQGIGYIMQYIAS

>SjMEG-8.1, C-terminus. GenBank: [AAW27175.1](https://www.ncbi.nlm.nih.gov/protein/AAW27175.1?report=genbank&log$=prottop&blast_rank=1&RID=X56E7ATX016)

IEIRKPKDVQQLGEKKTIMQKINDGFFYLFSEQEFHPLHDKSYLFNIWYLFKHSFFNLKNMRNLIFGS

>SjMEG-8.2, C-terminus. GenBank: [TNN07768.1](https://www.ncbi.nlm.nih.gov/protein/TNN07768.1?report=genbank&log$=prottop&blast_rank=1&RID=X56E7ATX016) & AAW25097.1KEKIVNGFNSIFGVEEFNPPKDSYFVDRLWLLFKHCFLNVKNVAKIFSK

>SjMEG-9 (complete). GenBank: [CAX69572.1](https://www.ncbi.nlm.nih.gov/protein/CAX69572.1?report=genbank&log$=prottop&blast_rank=1&RID=X56E7ATX016)

FVVQSNGKDEAKPEESQFFAIPFMMTIGSHLWSFLNGCFLDVENLKKLVFP

>SjMEG-11 (complete). GenBank: [CAX69679.1](https://www.ncbi.nlm.nih.gov/protein/CAX69679.1?report=genbank&log$=prottop&blast_rank=1&RID=X56E7ATX016)

DGEEQNPEPPRPRRKHPVLREVFLTAPQWLHIPFSLLGALASYAAYHFYG

>SjVAL7 (complete). GenBank: [AAW25717.1](https://www.ncbi.nlm.nih.gov/protein/AAW25717.1?report=genbank&log$=prottop&blast_rank=1&RID=X56E7ATX016)

VNWHIEDEEILALHNAYREAVKFGRVRDQPKAISMSKLQWSYQLAKLAENWTIHCIPKTSGLKFRNSSKWTYVGQNVAVVSKIRDAPAVWFNQHRNYNYTKNVCAAQKICADYKQLAYASTTHIGCAYKFCEKLNGTGKILVVCNYGPGGKFINRKPYQIFDYDDFYLY

>Sj irrelevant protein. GenBank:TNN14773.1

KVDGQPPAKYLGLSIVCNYGPGGDWNNEKPYIVKPPDECPKFQDVVSHNNHSSSVDHNPDASGSDRTHQNMSMSSNIRDAS

**ARRAY 1b**

As for 1a except:

>SjMEG-14, C-terminus. GenBank: [AAW27393.1](https://www.ncbi.nlm.nih.gov/protein/AAW27393.1?report=genbank&log$=prottop&blast_rank=1&RID=X56E7ATX016)

TPPTPTVTKRPHDERGVVAASIAPILIGLLGQGIGYIMHYIAI

>SjVAL7 (partial). GenBank: [AAW25717.1](https://www.ncbi.nlm.nih.gov/protein/AAW25717.1?report=genbank&log$=prottop&blast_rank=1&RID=X56E7ATX016)

KFGRVRDQPKAISMSKCNYGPGGKFINRKPYQ

>SjGST26. GenBank: [6JI6_A](https://www.ncbi.nlm.nih.gov/protein/6JI6_A?report=genbank&log$=prottop&blast_rank=1&RID=X56E7ATX016) and many others

MSPILGYWKIKGLVQPTRLLLEYLEEKYEEHLYERDEGDKWRNKKFELGLEFPNLPYYIDGDVKLTQSMAIIRYIADKHNMLGGCPKERAEISMLEGAVLDIRYGVSRIAYSKDFETLKVDFLSKLPEMLKMFEDRLCHKTYLNGDHVTHPDFMLYDALDVVLYMDPMCLDAFPKLVCFKKRIEAIPQIDKYLKSSKYIAWPLQGWQATFGGGDHPPK

**ARRAY 2**

>MEG-29. GenBank: [CAX69871.1](https://www.ncbi.nlm.nih.gov/protein/CAX69871.1?report=genbank&log$=prottop&blast_rank=1&RID=X56E7ATX016)

MIYVVKCGGEEEVPTYFMMTVFSIHLIQRIIWKFMF

>MEG-15. GenBank: [TNN09929.1](https://www.ncbi.nlm.nih.gov/protein/TNN09929.1?report=genbank&log$=prottop&blast_rank=1&RID=X56E7ATX016)

QRDPPKVHKEQHHQPIDYDALLSKFLRVLTEKVPQIPQIKNLPKEKVHKFITSLKKLIDEVHSLSLKTIDGKKP

>MEG-12. GenBank: no match

AVLTGVVGGETQNNEERQNEPSGFLRFLTYLYKSFKFACTFTNIMSWFTG

>Palmitoyl thioesterase. GenBank: [CAX69665.1](https://www.ncbi.nlm.nih.gov/protein/CAX69665.1?report=genbank&log$=prottop&blast_rank=1&RID=X56E7ATX016)

DNLPVVIWHGMGDHGTSIYIQYLSRVIKRIKPETYVKCITTNNSYIEDVRDTVFSSINEQLENVCRMISEDKNLSNGLHMIGISQGGVFVRALVQKCSLSNVGSVISIGGPQQGVFGVPHCIDEGFVPYCSWFHKLLSYVVYTKYIQNRVVQAQYWHDPLKEDVYRKYSQFLADINQEIQINETYRENMKKVQRLVLVKFSNDTVLIPKESEWFGFYRTGSLSDITRLQDSILYTEDRLGLQELDRRGDLHFIEKEGDHLQINSEWFVNNIVIPFLK

>Aspartyl protease. GenBank: [CAX72335.1](https://www.ncbi.nlm.nih.gov/protein/CAX72335.1?report=genbank&log$=prottop&blast_rank=2&RID=X56E7ATX016)

EIVRVPLHPVRRSSSWWRVSRNYFQPSFRVMTNGPTTRYKTTSEQLIDYRNLQYYGEVSVGTPPQRLRVLFDTGSTDTWFASRRCWFFDIFCWLFSFYDRTKSSTYRPDGSTFEVRYLDSNYSGIWSVDTVRINSLIIQNQAFAEMTNIFNWDYFTDKYDGIIGMSCRRISTYGNIPMFPNILANGVNMEPVFSFYLNRKEGVSIGGEMILGGVNPKYFKGDFEYIPTVSNHVWTIPMLSLRIKEMEFCNRCIATIDTGTSLIIGGKEQVDKINSLLGTWNSIFEKNVFDCSRIDMLPSIEFVFHKKKYILEPRHYVVKVSNLVGTICSSPFESIESLPPGIWILGDVFMGRFYTVYDFGQKRIGFANVSFV

>MEG-8.4. GenBank: [TNN06768.1](https://www.ncbi.nlm.nih.gov/protein/TNN06768.1?report=genbank&log$=prottop&blast_rank=1&RID=X56E7ATX016)

LTKESINYYKIQEKELKDINERDKKLKSMFRKFQENKQAKVVDEFQPDVNSTILNKLWQLFNHCFLTFDNLIRVIQNDHALL

>MEG-8.3. GenBank: [TNN07769.1](https://www.ncbi.nlm.nih.gov/protein/TNN07769.1?report=genbank&log$=prottop&blast_rank=1&RID=X56E7ATX016)

VAIPAAPKKNESLSFKEKIMDMYNHWVNQDEYNPPKDSEFYERFWLLFKHCFMNSKKLAKIIPFIG

>Phospholipase A #1. GenBank: TNN19273.1

IPLLTVINKNNTNAKYPIILIPGLGGTQAYCESTINDEKKSFTPFNVWLNFLYILLPYKGFEYFRLKHDPHTYESYDVNECNVTFPGWGDTWSVEYLSQHISYEYFAPIVSEFIKDAFYVRNFTLRGAPYDFRKSPNDNKLFVVKLRKLIEETYKNGFNRPVVLLGHSMGSIYTLHFLKHQTKRWKQKYIKSFISVSAPFGGTVETLHVLTSGDNFGIFLRSPLPYRDLFRTMTSIIATLPNPKLWSKDEVLIVTPHRNYTVLDYPQYFSDADYLTGYNLFTRFISSFDPLEPPKDVPEVYCIYGSGLLSVEQIIFQSSSLFVSSFPNQSPRIIYGNGDGTVNLRSLQVCKKWPSVKVVEISVSGHRQILNEKRFIDFLKYHVNT

>MEG-19. GenBank: [TNN08578.1](https://www.ncbi.nlm.nih.gov/protein/TNN08578.1?report=genbank&log$=prottop&blast_rank=1&RID=X56E7ATX016)

AGILWNNNPVMRRENISIVPITEIQGPFVVHENRYTLVRKPKLVCKQKHGVIKCYVNEGTHVQHFNGKILLWSLLTINFVYLFLSS

>MEG-26. GenBank: no match

EGVIFDVELFILDLWTNFYQRLEETIQCFLAVLPKQLGGSSKACY

>Phospholipase A #2. GenBank: [TNN19274.1](https://www.ncbi.nlm.nih.gov/protein/TNN19274.1?report=genbank&log$=prottop&blast_rank=1&RID=X56E7ATX016)

GPIKNAPLQFLTKDNNTYPLILIPGLGGSQAYCQFNATKSVDTLVWLNLFYMAIPEKLQGYFGLHYNPVTLNAEDNPECHVVFPGWGDTKSVEYLQTKGFKFFNYFGSLINAITKNKFFIKNFTIRGAPFDFRKLPNENIHFMDKLKLLVEETYTNAHQRPVVLLGHSMGSLYTLNFLNKQTKQWKQKYIRSYISVSAPFGGAVKALIAIVTGDNFGIFYRSPLAFRKALRSFPSIIANLPDPRIWPSNDVLIATPLKNYTAQDYLALFKDIDFPLGYQVMQKALHEFTTLEYPKDVPEVYCVYSSGLLTMKRLIYKSPGLFRSKFPNQSPVLQYEDGDGTVNLHSLQYCNKWPNSSLVHLIASNHVPILRDERFIKFVKERISNDFVN

>MEG-22. GenBank: no match

KETQLLPPLVVHEKLMTNKENQKLIKRRPLIKRYPMPIPHPPVYFQEPTFSFENFTFTSLVHYIIKKALWIFDNLMLKGFENEIKHPNASEMYKFRLATKSNLK

**ARRAY 3**

>Tetraspanin_e81080. GenBank: [TNN11204.1](https://www.ncbi.nlm.nih.gov/protein/TNN11204.1?report=genbank&log$=prottop&blast_rank=1&RID=X56E7ATX016)

AVWNQKSTLLNVYQILDYYKECRFPKFDPVDNYDNIIYKGLSLPIPCCRMNDNLEIINTTCPFQYTIFNSNIGRDCSKLFAEEISSYTDTFI

>Tetraspanin_e15313. GenBank: TNN06593.1

TAQTILSKFNTILQNPAPVIAVMHDQFYAATNHSLLNAVKFFYAKPQYEIELDQLQTDFKCCGAKSYMDYRKLAVNIPFTCLVGHLVYARGCIEVLSDYIQQYII

>Annexin_e53714. GenBank: [AAW26872.1](https://www.ncbi.nlm.nih.gov/protein/AAW26872.1?report=genbank&log$=prottop&blast_rank=1&RID=X56E7ATX016)

MLLDSSVNVYYVLAEIIIMLLQLYDKQTPPFSRSTLHNINTEGILYEPTVVSSPGFSPENDVYKIGNMTRDEKNDKLLEVLLSRTNAERQSIVRNYQKLFNKSILLEISDINMRSMKLFIQDMLTDTSMLLADELNKAMKTSDLQLVTSILIDFWGDEFNQVESVYRIYSNESIWQHINNSFGVTVKNILRCTVMTRKHEQKLDYPIKGKGGKRIVETGLVTKIHEILTRKLATKEYTAQSLEKLFCLLYPFEMEMLNKQFNEKTTGKDLSAFIENNTNGLMRDVLIAMLNHSVNKPMYFATIIHDAIHKNQTNTSTVHRLLISRSEIDLYTINKVYKVQYGKYLLNDIKKEFNGVYDDVLTRPLRKPELSDSVQHSIPITFYEG

>Annexin_AAX26603_partial. GenBank: [AAX26603.2](https://www.ncbi.nlm.nih.gov/protein/AAX26603.2?report=genbank&log$=prottop&blast_rank=1&RID=X56E7ATX016)

IYSNKSIWQDIEHHFGEPVKNILYCTVDSRKHELKMEHPNKGRGGKPIVNRARIISLFRILTRILDSKKDVQQSLGELLCFLDPFELEMLNKQFKKKPVKIYQQSFRTKHLVKRMMC

>Cystatin_d19034. GenBank: [TNN18702.1](https://www.ncbi.nlm.nih.gov/protein/TNN18702.1?report=genbank&log$=prottop&blast_rank=1&RID=X56E7ATX016)

FNTGGFQQINQYGRHSLNQSVIQQSVVLANKMFNSLHWFTSNDVTNTTTQIVAGLMFRYNLHLVQTNCTKKSVFKHVSNTNGSKCRRDNKTAGAVCRVQVLYQPWEKNEYDIKIISCQSKVEPSSKKENRLNKPIRRYTNATSMVKHYNI

>DUF4321_e14800_Natterin. GenBank: [TNN12829.1](https://www.ncbi.nlm.nih.gov/protein/TNN12829.1?report=genbank&log$=prottop&blast_rank=1&RID=X56E7ATX016)

FCPVGAITVDNVCVARCKHSGELLPGKLVPMNGKCYCSYGGAEIESYNYEVLCESFIPGSCRGYCWETAYDGDVPKNAIVAGIAKDGQPLYIVKGSVNGETCFGKLHEGHSCAYLPWGGKEHSVSEYDVLVWQKY

>MEG-n.1_e80567. GenBank: no match

NNTTTNMTTQTVESKSSGIWDVLRTFFTSLCTFFSFWKTINSFFGFV

>MEG-n.2_e29716_EST_FL657167. GenBank: no match

TTETNDSSTSTSTSQETTQSVTQQSSIWDTIRTILTTICTLFSFWESIRGIFNFG

>MEG-26.2_e5041_h14719. GenBank: no match

SGDTGDAVIFNFRLFFLGIWDNLCCRLHGTFNYFLDDLRKRFGGSRG

>MEG-26.4_hybrid_c10831_e106652. GenBank: no match

EAIFDLKLFILDFWTNLGKRLGETLKYFIKNWNGPFTQGGGLIFDMVTFIVNIWLNLSDRLRGTFGCFLDVLDPQLGGKNKS

>MEG-26.6_e103685. GenBank: no match

DVIFNLEMFILDTWTNLCNRLAGTFRCLLEILPVTLGGKDCICITANKTSCKKNK

>MEG-26.5_e104245. GenBank: no match

EGTIFDLVTFIVKLWDNFGLRLRDTFQCFLDALPKTLGGKNESCKV

.
