# Supplementary material for: Mapping the epitopes of Schistosoma japonicum esophageal gland proteins for incorporation into vaccine constructs
Source: PLoS One. 2020 Feb 27;15(2):e0229542. doi: 10.1371/journal.pone.0229542 (PMC7046203; doi:10.1371/journal.pone.0229542)
Supplement: S3 Fig — A. The amino acid sequences are concatenated from Table 1. VAL-7 is represented by an epitope predicted by BepiPred. B, The nucleotide sequence of the synthetic gene for splicing into the expression vector, and its optimised version for expression in E. coli. C, Heat map of the reactivity of sera from two rabbits immunised with the recombinant protein, mapped onto Array 1a. The regions on the array represented by the protein are colour-coded. (PPTX) [file pone.0229542.s003.pptx]

## Slide 1
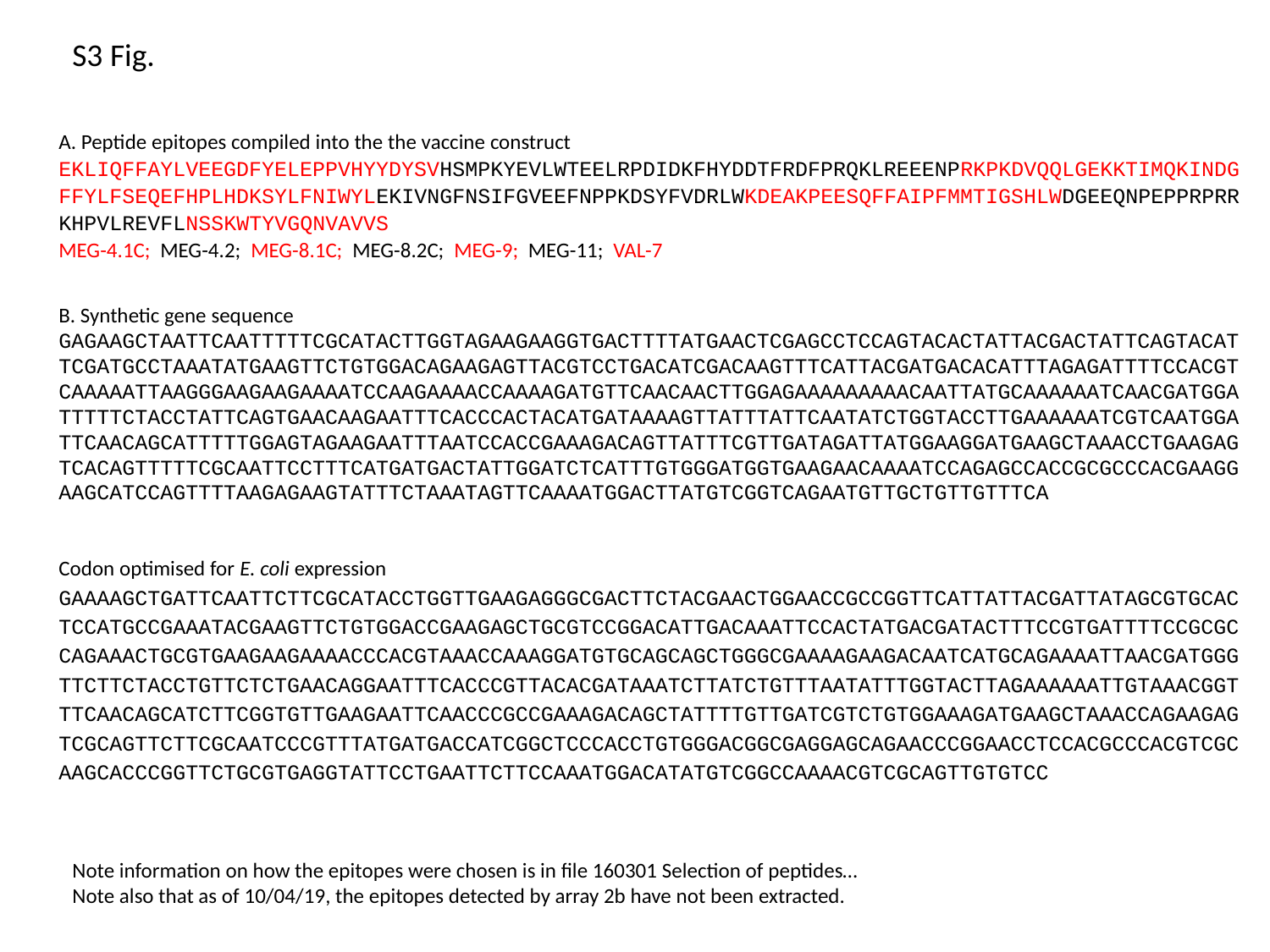

S3 Fig.
A. Peptide epitopes compiled into the the vaccine construct
EKLIQFFAYLVEEGDFYELEPPVHYYDYSVHSMPKYEVLWTEELRPDIDKFHYDDTFRDFPRQKLREEENPRKPKDVQQLGEKKTIMQKINDGFFYLFSEQEFHPLHDKSYLFNIWYLEKIVNGFNSIFGVEEFNPPKDSYFVDRLWKDEAKPEESQFFAIPFMMTIGSHLWDGEEQNPEPPRPRRKHPVLREVFLNSSKWTYVGQNVAVVS
MEG-4.1C; MEG-4.2; MEG-8.1C; MEG-8.2C; MEG-9; MEG-11; VAL-7
B. Synthetic gene sequence
GAGAAGCTAATTCAATTTTTCGCATACTTGGTAGAAGAAGGTGACTTTTATGAACTCGAGCCTCCAGTACACTATTACGACTATTCAGTACATTCGATGCCTAAATATGAAGTTCTGTGGACAGAAGAGTTACGTCCTGACATCGACAAGTTTCATTACGATGACACATTTAGAGATTTTCCACGTCAAAAATTAAGGGAAGAAGAAAATCCAAGAAAACCAAAAGATGTTCAACAACTTGGAGAAAAAAAAACAATTATGCAAAAAATCAACGATGGATTTTTCTACCTATTCAGTGAACAAGAATTTCACCCACTACATGATAAAAGTTATTTATTCAATATCTGGTACCTTGAAAAAATCGTCAATGGATTCAACAGCATTTTTGGAGTAGAAGAATTTAATCCACCGAAAGACAGTTATTTCGTTGATAGATTATGGAAGGATGAAGCTAAACCTGAAGAGTCACAGTTTTTCGCAATTCCTTTCATGATGACTATTGGATCTCATTTGTGGGATGGTGAAGAACAAAATCCAGAGCCACCGCGCCCACGAAGGAAGCATCCAGTTTTAAGAGAAGTATTTCTAAATAGTTCAAAATGGACTTATGTCGGTCAGAATGTTGCTGTTGTTTCA
Codon optimised for E. coli expression
GAAAAGCTGATTCAATTCTTCGCATACCTGGTTGAAGAGGGCGACTTCTACGAACTGGAACCGCCGGTTCATTATTACGATTATAGCGTGCACTCCATGCCGAAATACGAAGTTCTGTGGACCGAAGAGCTGCGTCCGGACATTGACAAATTCCACTATGACGATACTTTCCGTGATTTTCCGCGCCAGAAACTGCGTGAAGAAGAAAACCCACGTAAACCAAAGGATGTGCAGCAGCTGGGCGAAAAGAAGACAATCATGCAGAAAATTAACGATGGGTTCTTCTACCTGTTCTCTGAACAGGAATTTCACCCGTTACACGATAAATCTTATCTGTTTAATATTTGGTACTTAGAAAAAATTGTAAACGGTTTCAACAGCATCTTCGGTGTTGAAGAATTCAACCCGCCGAAAGACAGCTATTTTGTTGATCGTCTGTGGAAAGATGAAGCTAAACCAGAAGAGTCGCAGTTCTTCGCAATCCCGTTTATGATGACCATCGGCTCCCACCTGTGGGACGGCGAGGAGCAGAACCCGGAACCTCCACGCCCACGTCGCAAGCACCCGGTTCTGCGTGAGGTATTCCTGAATTCTTCCAAATGGACATATGTCGGCCAAAACGTCGCAGTTGTGTCC
Note information on how the epitopes were chosen is in file 160301 Selection of peptides…
Note also that as of 10/04/19, the epitopes detected by array 2b have not been extracted.
