# Supplementary material for: Mapping the epitopes of Schistosoma japonicum esophageal gland proteins for incorporation into vaccine constructs
Source: PLoS One. 2020 Feb 27;15(2):e0229542. doi: 10.1371/journal.pone.0229542 (PMC7046203; doi:10.1371/journal.pone.0229542)
Supplement: S4 Fig — (PPTX) [file pone.0229542.s004.pptx]

## Slide 1
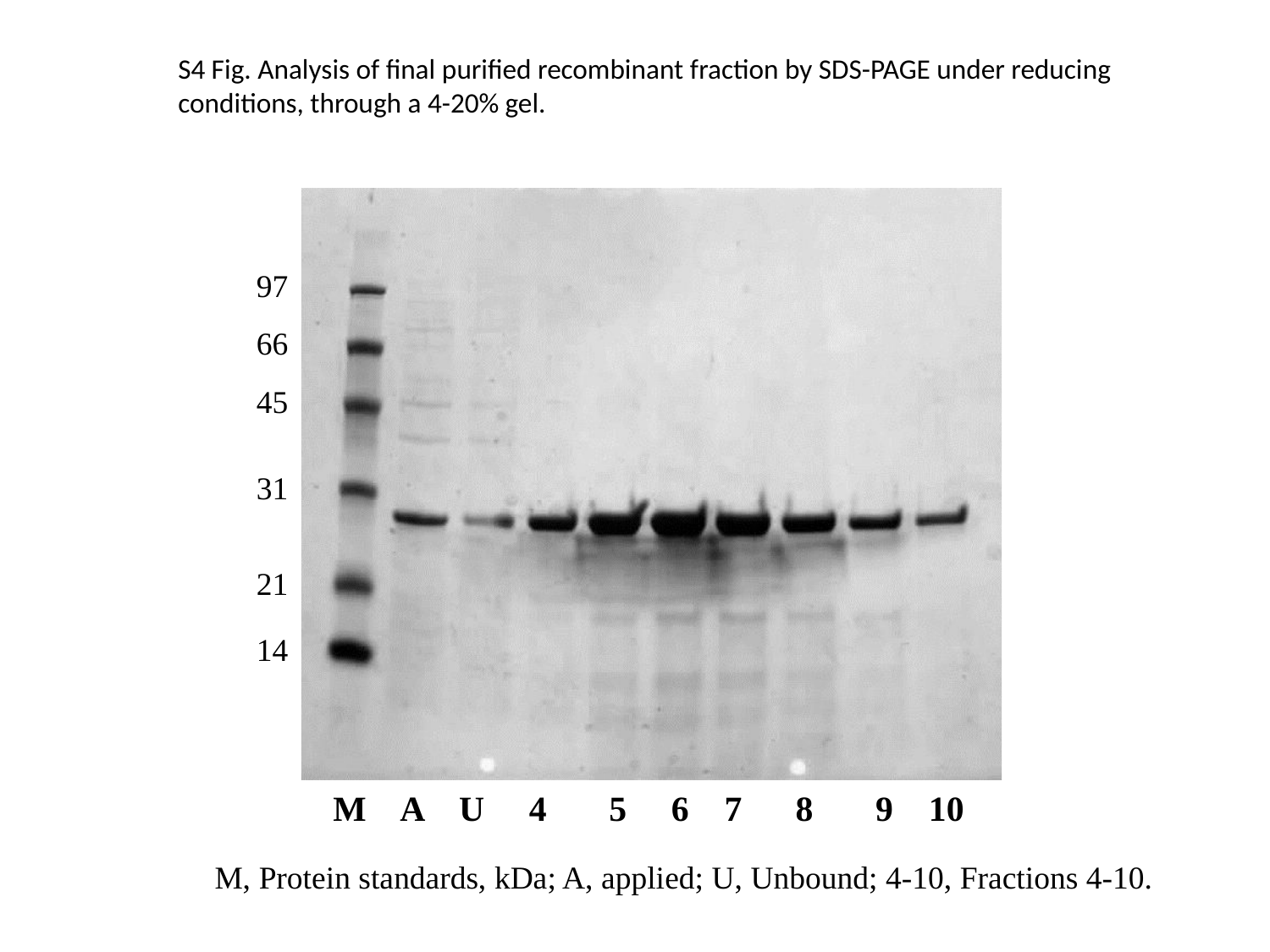

S4 Fig. Analysis of final purified recombinant fraction by SDS-PAGE under reducing conditions, through a 4-20% gel.
M A U 4 5 6 7 8 9 10
97
66
45
31
21
14
M, Protein standards, kDa; A, applied; U, Unbound; 4-10, Fractions 4-10.
