# Supplementary material for: Mapping the epitopes of Schistosoma japonicum esophageal gland proteins for incorporation into vaccine constructs
Source: PLoS One. 2020 Feb 27;15(2):e0229542. doi: 10.1371/journal.pone.0229542 (PMC7046203; doi:10.1371/journal.pone.0229542)
Supplement: S1 Table — A. Epitopes selected from Array 2. B. Concatenated amino acids for a putative artificial protein sequence. (PPTX) [file pone.0229542.s005.pptx]

## Slide 1
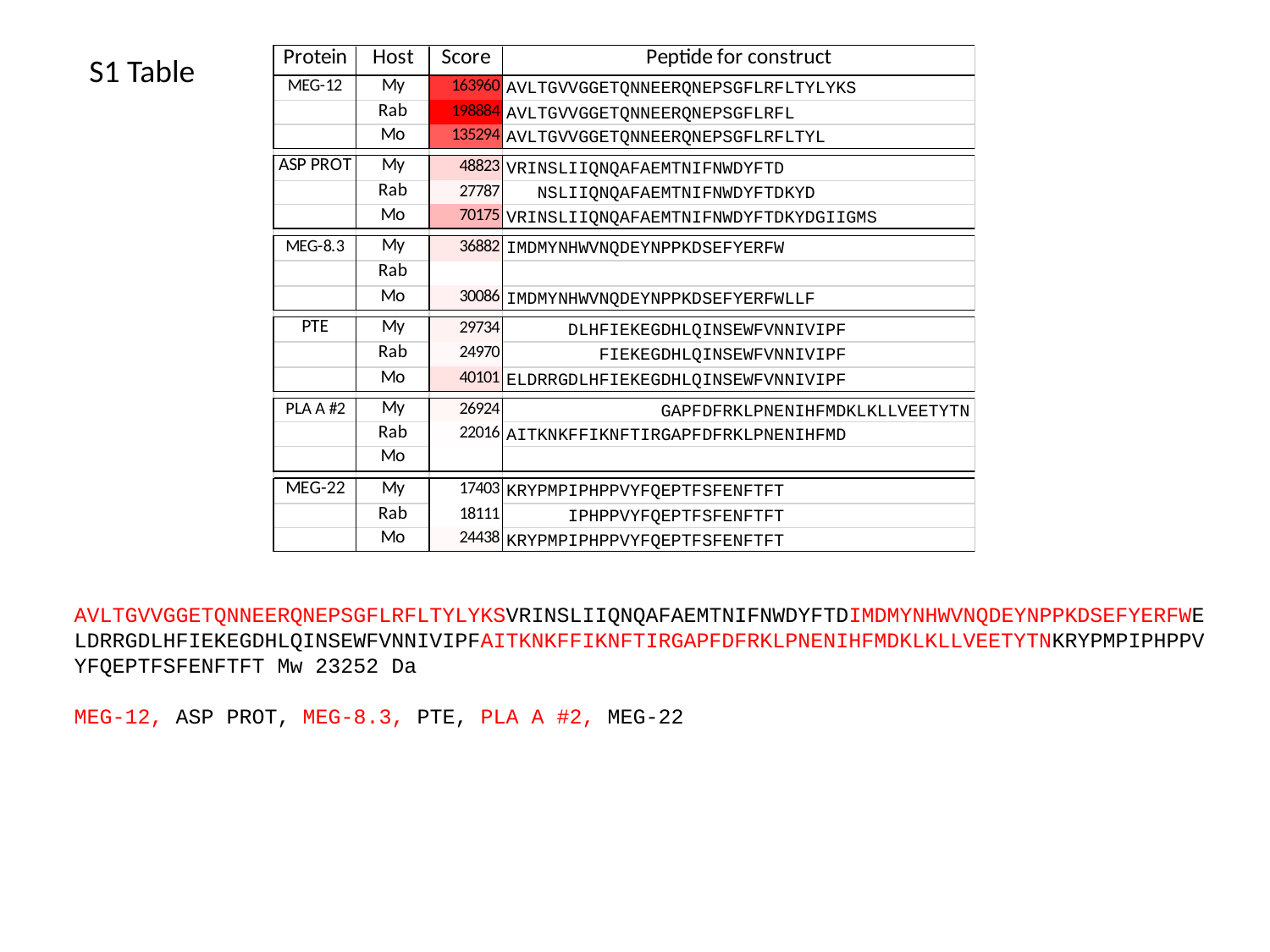

S1 Table
AVLTGVVGGETQNNEERQNEPSGFLRFLTYLYKSVRINSLIIQNQAFAEMTNIFNWDYFTDIMDMYNHWVNQDEYNPPKDSEFYERFWELDRRGDLHFIEKEGDHLQINSEWFVNNIVIPFAITKNKFFIKNFTIRGAPFDFRKLPNENIHFMDKLKLLVEETYTNKRYPMPIPHPPVYFQEPTFSFENFTFT Mw 23252 Da
MEG-12, ASP PROT, MEG-8.3, PTE, PLA A #2, MEG-22
