# Supplementary material for: Mapping the epitopes of Schistosoma japonicum esophageal gland proteins for incorporation into vaccine constructs
Source: PLoS One. 2020 Feb 27;15(2):e0229542. doi: 10.1371/journal.pone.0229542 (PMC7046203; doi:10.1371/journal.pone.0229542)
Supplement: S2 Table — A. Epitopes selected from Array 3. B. Concatenated amino acids for a putative artificial protein sequence. (PPTX) [file pone.0229542.s006.pptx]

## Slide 1
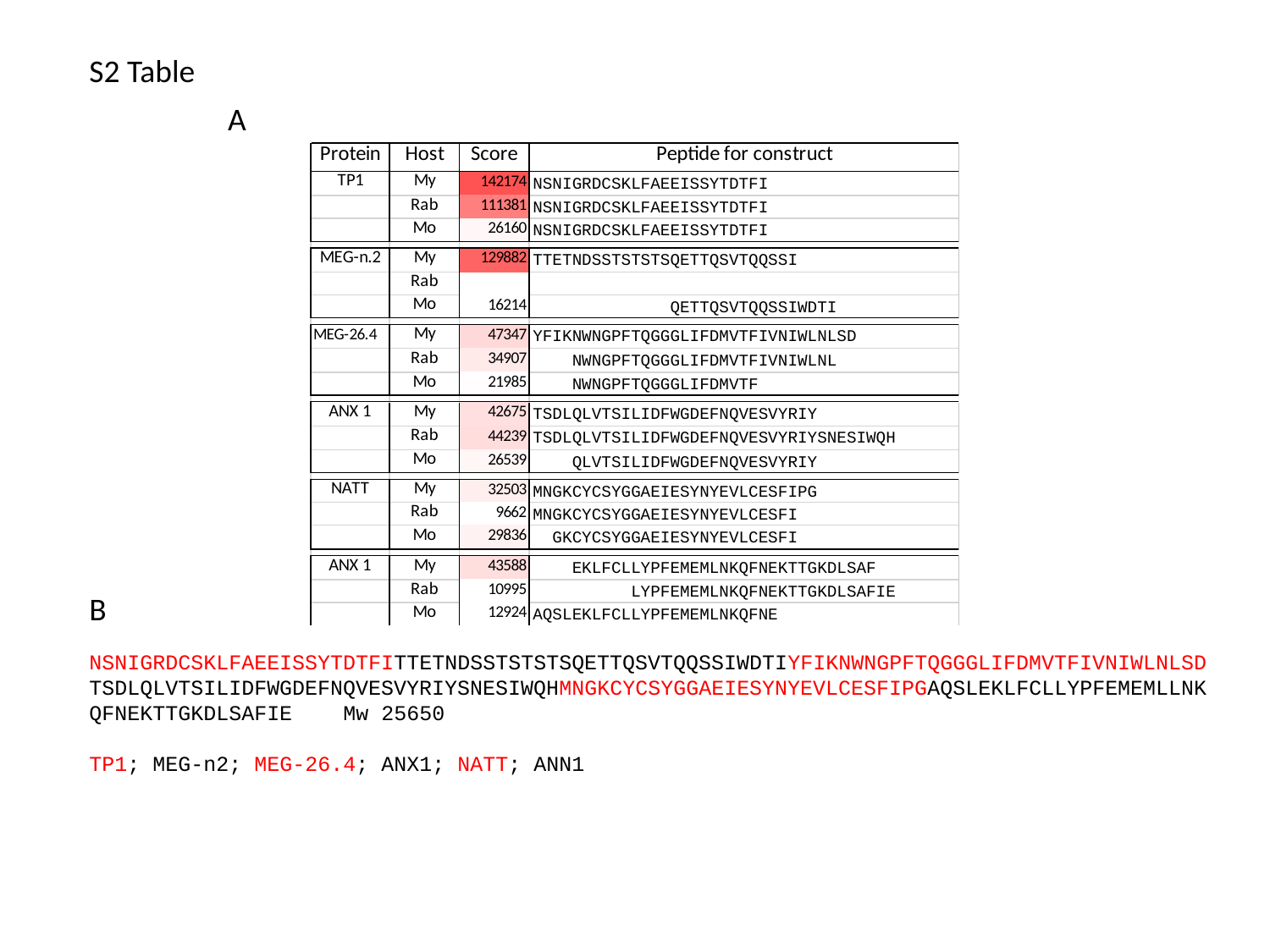

S2 Table
A
B
NSNIGRDCSKLFAEEISSYTDTFITTETNDSSTSTSTSQETTQSVTQQSSIWDTIYFIKNWNGPFTQGGGLIFDMVTFIVNIWLNLSDTSDLQLVTSILIDFWGDEFNQVESVYRIYSNESIWQHMNGKCYCSYGGAEIESYNYEVLCESFIPGAQSLEKLFCLLYPFEMEMLLNKQFNEKTTGKDLSAFIE	Mw 25650
TP1; MEG-n2; MEG-26.4; ANX1; NATT; ANN1
